# Additive Anticancer and Antioxidant Effects of Metformin and Luteolin in Breast and Colorectal Cancer Cell Lines

**DOI:** 10.3390/ph18111660

**Published:** 2025-11-01

**Authors:** Katarzyna Gębczak, Łucja Cwynar-Zając, Monika Sapeta-Nowińska, Ewa Barg

**Affiliations:** 1 Department of Basic Medical Sciences and Immunology, Division of Basic Medical Sciences, Faculty of Pharmacy, Wroclaw Medical University, 50-556 Wroclaw, Poland; 2 Department of Biochemistry, Molecular Biology and Biotechnology, Faculty of Chemistry, Wroclaw University of Science and Technology, 50-370 Wroclaw, Poland

**Keywords:** cancer cell lines, cell culture, metformin, luteolin, oxidative stress, metabolomics, ^1^HNMR

## Abstract

**Background/Objectives**: Metformin (Met) is a potent antidiabetic drug that also exhibits anticancer, antioxidant, and organ-protective properties. Luteolin (Lut), a naturally occurring flavonoid found in many plant species, possesses anticancer, antioxidant, and anti-inflammatory effects. Since both compounds affect cellular metabolism and oxidative balance, the analysis of metabolites produced in living cells provides insight into biochemical alterations occurring in cancer cells and enables monitoring of treatment response. **Methods**: In this study, Met (1–20 mM) and Lut (1–100 µM) were tested in vitro, both individually and in combination, to evaluate their effects on cell viability, free radical levels, and metabolite profile alterations in cancer and normal cell lines (MDA-MB-231, SW620, and V79). Cell viability was assessed using the MTT assay at two time points (24 h and 48 h), while reactive oxygen species (ROS) levels were measured after hydrogen peroxide stimulation (100 µM H_2_O_2_) using the DCF-DA assay. Metabolomic changes induced by Met and Lut were analyzed by ^1^H NMR spectroscopy. **Results**: The analysis showed that Lut reduced the viability of MDA-MB-231 cells at both time points, whereas Met decreased viability only after prolonged incubation. Met did not inhibit the proliferation of SW620 colorectal cancer cells, while Lut reduced viability at higher concentrations (100 µM after 24 h, and 50–100 µM after 48 h). **Conclusions**: The combination of metformin and luteolin demonstrated additive effects in reducing cell viability and oxidative stress compared with single-compound treatments. Normal V79 fibroblasts responded to both Met and Lut, individually and in combination. Both compounds exhibited moderate antioxidant properties in cells exposed to 100 µM H_2_O_2_. Lut (25 µM) reduced free radical levels in MDA-MB-231 cells, whereas Met (2.5 mM) did so in SW620 cells. The combination of both compounds increased ROS levels in SW620 cells subjected to oxidative stress. Overall, co-treatment with metformin and luteolin altered metabolic pathways and induced changes in intra- and extracellular metabolite levels across all tested cell lines. The observed additive effects suggest that the combined use of metformin and luteolin may enhance anticancer and antioxidant responses, warranting further in vivo studies to confirm these interactions.

## 1. Introduction

The growth and development of cancer cells begin with the initiation process associated with genetic mutations, followed by promotion and progression. Studies have confirmed an increased risk of liver, pancreatic, endometrial, colorectal, kidney, bladder, and breast cancers in patients with diabetes [1,2]. A relationship has been identified between elevated levels of endogenous insulin and the development of pancreatic and liver cancer. Hyperinsulinemia may indirectly promote tumor growth by increasing insulin-like growth factor (IGF) levels, directly stimulating insulin receptors expressed on cancer cells, and activating chronic inflammation. This leads to the modification of signaling pathways that may enhance proliferation and inhibit apoptosis in cancer cells [3]. An indirect effect of hyperinsulinemia on the reduced hepatic synthesis of sex-hormone-binding globulin (SHBG) has also been demonstrated, resulting in elevated levels of steroid sex hormones. A decrease in serum SHBG levels has been observed in obese women with polycystic ovary syndrome, and elevated endogenous sex hormone levels are associated with an increased risk of breast cancer in postmenopausal women [4,5]. Chronic hyperglycemia is also linked to increased levels of reactive oxygen species (ROS), while prolonged oxidative stress contributes to cellular damage and may promote the development of diabetes and its complications [6]. On the other hand, chronic inflammation induced by hyperinsulinemia can destabilize redox processes, leading to the initiation and progression of various cancers [7]. Hyperglycemia may further create an environment favorable to tumor development due to the high energy demand and glucose uptake of cancer cells.

Metformin has become the most commonly prescribed and effective drug for patients with type 2 diabetes mellitus (T2DM), and, in 2011, it was included in the World Health Organization’s (WHO) List of Essential Medicines [8]. The antidiabetic effect of metformin is primarily based on the reduction of hepatic glucose metabolism rather than on increased insulin secretion or improved pancreatic beta-cell function [9]. The inhibition of hepatic gluconeogenesis and glucose production is regulated by acetyl-coenzyme A (acetyl-CoA) and substrate-dependent [NADH]:[NAD^+^] redox processes. Metformin inhibits the conversion of glycerol 3-phosphate (G3P) to dihydroxyacetone phosphate (DHAP), thereby impairing gluconeogenesis from glycerol. Glycerol is phosphorylated to G3P and converted to DHAP via glycerol-3-phosphate dehydrogenase 2 (GPD2), which is required for the entry of glycerol into the gluconeogenic pathway. Similarly, an increased cytosolic [NADH]:[NAD^+^] ratio inhibits lactate dehydrogenase, reducing gluconeogenesis from lactate. In contrast, substrates such as pyruvate, DHAP, and alanine are redox independent and are not inhibited by metformin, indicating the substrate-selective action of the drug. Metformin also inhibits mitochondrial respiratory chain complex I in hepatocytes, skeletal muscle, endothelial cells, and pancreatic beta cells. Complex I serves as the site of NADH oxidation and proton transfer in the mitochondrial chain, generating energy for gluconeogenesis and hepatic glucose production. Reduced electron transport decreases the [ATP]:[ADP] and [ATP]:[AMP] ratios, lowering the cellular energy state and indirectly reflecting the metabolic impact of metformin [10]. Traditionally used for the treatment of type 2 diabetes, metformin has recently gained attention as a potential anticancer agent. Epidemiological studies indicate that patients with type 2 diabetes treated with metformin have a lower risk of developing cancer and reduced cancer-related mortality. Metformin may inhibit complex I of the mitochondrial respiratory chain, thereby lowering ATP levels and influencing overall energy metabolism. Another anticancer role attributed to metformin involves its effects on tumor characteristics, including regulation of the cell cycle, apoptosis, cancer cell migration, invasion, metastasis, metabolism, and immunity [11,12]. Ongoing research is exploring the anticancer potential of metformin as a therapeutic option for specific cancers, as well as its role in the aging process in healthy individuals [13]. Metformin demonstrates significance beyond diabetes management by improving immunotherapy outcomes, delaying multimorbidity, and reversing biological markers of aging—influencing the characteristic hallmarks of aging and cancer biology [14,15]. Luteolin (3,4,5,7-tetrahydroxyflavone) and its glycosides are among the most common flavonoids found in edible plants and are widely distributed in species used in traditional medicine. It has been identified in numerous vegetables and fruits, including celery, bell peppers, apple peels, cabbage, carrots, broccoli, and parsley, as well as in certain flowers, herbs, and spices [16,17]. Luteolin possesses multiple biological activities, including antioxidant, anti-inflammatory, anticancer, and antimicrobial properties [17,18,19,20]. Preclinical studies have shown its ability to induce apoptosis, inhibit cell proliferation and angiogenesis, prevent carcinogenesis, and suppress tumor growth in animal models [21,22,23]. These findings suggest that luteolin has both chemopreventive and chemotherapeutic potential. It is also believed to help prevent several diseases, particularly cardiovascular disorders, diabetes, and neurodegenerative conditions [24]. The antidiabetic effect of luteolin may be related to its antioxidant capacity, which improves redox balance in the body [16]. Studies further indicate that luteolin can inhibit glycolysis and ATP production in cancer cells in vitro [25] and suppress angiogenesis in vitro and in vivo at low micromolar concentrations [26]. Recent evidence suggests that luteolin may sensitize cancer cells to the cytotoxic effects of certain chemotherapeutic drugs. For instance, luteolin-induced ROS accumulation plays a key role in suppressing the nuclear transcription factor NF-κB and enhancing Jun N-terminal kinase (JNK) activation, thereby sensitizing cancer cells to tumor necrosis factor-alpha (TNF-α)-induced apoptosis [27,28]. Additionally, luteolin enhances the anticancer activity of cisplatin in vitro and in vivo by stabilizing and accumulating p53 [29].

Metabolomics is a rapidly developing field driven by advances in bioinformatics. It focuses on the study of the composition and quantity of small-molecule compounds, or metabolites, present in cells, biological fluids, or tissues. Metabolomic profiling tracks changes in the metabolome as a response of the organism to drugs or disease progression [30].

Analytical methods such as mass spectrometry (MS), nuclear magnetic resonance spectroscopy (NMR), liquid chromatography (LC), and gas chromatography (GC) are used, and the resulting data are analyzed bioinformatically. A study by Ser et al. demonstrated that treatment with 5-fluorouracil (5-FU), a commonly used chemotherapeutic drug, results in increased production of the deoxyuridine nucleotide—which may serve as a potential biomarker of a positive response to 5-FU therapy [31]. Metabolomic analysis has enabled the identification of clinically relevant biomarkers, such as taurine, hypotaurine, glutamate, and aspartate pathways, recognized in early breast cancer diagnosis. Additionally, acetylcarnitine has been identified as a potential biomarker. Biomarkers can be classified according to the disease stage as prognostic, diagnostic, or tumor markers. Furthermore, they may be divided into global biomarkers—those present across multiple cancer types—and those specific to a particular tumor type [32].

In recent years, the study of natural compounds as key therapeutic targets has yielded promising results in cancer treatment. The combination of luteolin with other clinical drugs, such as sorafenib in hepatocellular carcinoma (HCC) and lapatinib in HER-2+ breast cancer cells, highlights the therapeutic relevance of this approach [33]. Previous studies have suggested that combinations of natural compounds may exert additive or complementary effects, enhancing anticancer efficacy. The rationale for combining metformin and luteolin stems from their complementary mechanisms of action. Metformin primarily modulates cellular metabolism through AMPK activation and inhibition of mitochondrial complex I, leading to reduced oxidative stress and energy depletion in cancer cells. Luteolin, in turn, exerts antioxidant and pro-apoptotic effects via ROS regulation, modulation of the AKT/mTOR signaling pathway, and induction of mitochondrial dysfunction. Based on these properties, it was hypothesized that their combined use could produce additive effects by targeting both metabolic and oxidative pathways involved in cancer cell survival. Similar additive or cooperative effects of metformin with flavonoids such as quercetin or resveratrol have been reported in previous studies, supporting the rationale for investigating this combination [34,35,36].

This study aimed to evaluate the combined effects of luteolin and metformin on metabolic processes, antioxidant activity, and metabolomic alterations in MDA-MB-231 (breast cancer), SW620 (colorectal cancer), and V79 (fibroblast) cell lines. The MDA-MB-231 cell line (triple-negative breast cancer) was selected to represent an aggressive breast carcinoma subtype, while SW620 cells were chosen as a model of colorectal adenocarcinoma. Both metformin and luteolin have previously demonstrated cytotoxic and antiproliferative activity in these cell types. Metformin has been shown to inhibit proliferation and induce apoptosis in MDA-MB-231 and SW620 cells [37,38], whereas luteolin exhibits strong antioxidant and pro-apoptotic effects in both MDA-MB-231 and SW620 cells [33,39,40]. These well-characterized cancer models were therefore selected to enable comparative assessment of the compounds’ effects across distinct tumor types and phenotypes. An inflammatory condition was induced using 0.1 mM hydrogen peroxide (H_2_O_2_). The SW620 cell line was derived from colorectal adenocarcinoma tissue of a 51-year-old Caucasian male (Dukes C stage colorectal cancer) [Online] Available: https://www.atcc.org/products/ccl-227 (accessed on 2 April 2024). The V79 cell line originates from the lung tissue of a young male Chinese hamster and exhibits fibroblast-like morphology (“ATCC Animal Cells V79”, [Online]. Available: https://www.atcc.org/products/ccl-93 (accessed on 4 May 2024)). The MDA-MB-231 cell line was established from mammary gland adenocarcinoma of a 40-year-old woman and displays epithelial-like morphology. Cancer-related markers expressed in this line include epidermal growth factor (EGF) and transforming growth factor beta (TGF-β) (“ATCC Human Cells MDA”, [Online]. Available: https://www.atcc.org/products/crm-htb-26 (accessed on 4 May 2023)).

## 2. Results

### 2.1. Effect of Luteolin and Metformin on Viability of SW620, MDA-MB-231, and V79 Cells

The MTT assay was employed to evaluate the effects of metformin (1–20 mM) and luteolin (1–100 µM) on the viability of normal fibroblast cells (V79) and cancer cell lines (SW620 and MDA-MB-231). Cell viability was measured at two time points: after 24 h and 48 h of incubation with the tested compounds.

**Figure 1:**
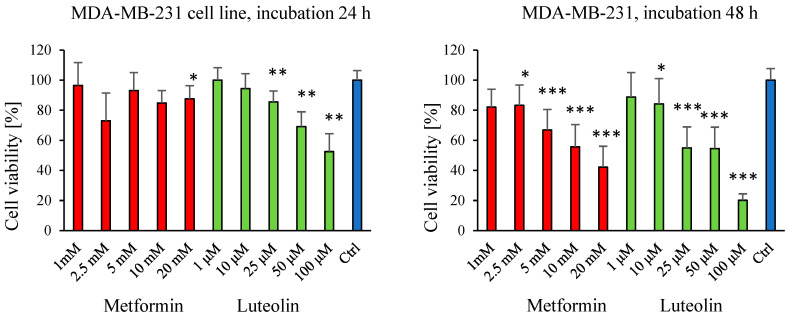
Effect of luteolin (1–100 μM) and metformin (1–20 mM) on the viability of MDA-MB-231 cells, assessed by the MTT assay. Absorbance values of cells treated with luteolin or metformin (E) are expressed relative to untreated control cells (E_0_) after 24 h and 48 h of incubation. Each bar represents the mean ± SD from three independent experiments. * *p* ≤ 0.05; ** *p* < 0.01; *** *p* < 0.001.

**Figure 2:**
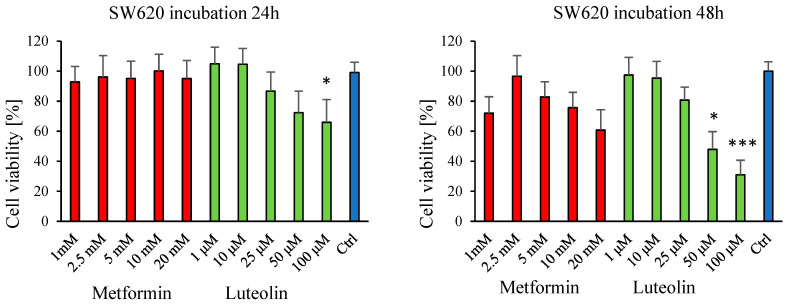
Effect of luteolin (1–100 μM) and metformin (1–20 mM) on the viability of SW620 cells, assessed by the MTT assay. Absorbance values of cells treated with luteolin or metformin (E) are expressed relative to untreated control cells (E_0_) after 24 h and 48 h of incubation. Each bar represents the mean ± SD from three independent experiments. * *p* ≤ 0.05; *** *p* < 0.001.

In both the breast cancer cell line (MDA-MB-231) and the colorectal cancer cell line (SW620), a distinct dose-dependent decrease in cell viability was observed after 24 h of exposure to luteolin, corresponding to increasing concentrations. Prolonging the incubation period to 48 h resulted in a significant inhibition of cell proliferation following treatment with both luteolin and metformin (Figure 1 and Figure 2).

**Figure 3:**
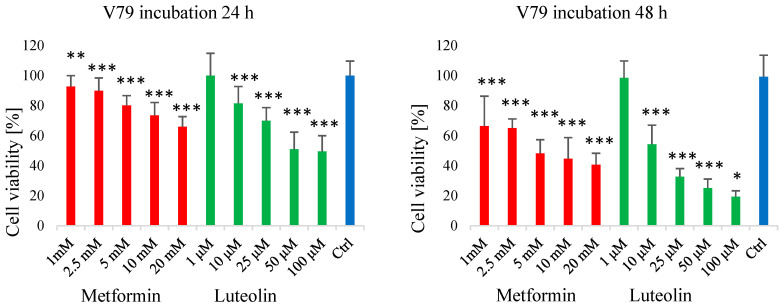
Effect of luteolin (1–100 μM) and metformin (1–20 mM) on the viability of V79 cells, assessed by the MTT assay. Absorbance values of cells treated with luteolin or metformin (E) are expressed relative to untreated control cells (E_0_) after 24 h and 48 h of incubation. Each bar represents the mean ± SD from three independent experiments. * *p* ≤ 0.05; ** *p* < 0.01; *** *p* < 0.001.

A pronounced reduction in the viability of V79 fibroblasts was also observed in response to both compounds, depending on the incubation time, except for luteolin at the concentration of 1 µM (Figure 3).

In the subsequent MTT assay, the effects of selected concentrations of metformin (2.5 mM) and luteolin (10 µM and 25 µM) were evaluated after 48 h of incubation. The impact of the compounds applied separately and in combination was assessed using two mixtures: MIX 1 (metformin 2.5 mM + luteolin 10 µM) and MIX 2 (metformin 2.5 mM + luteolin 25 µM). The results were normalized to untreated control cells (100% viability), and three independent experiments were performed.

**Figure 4:**
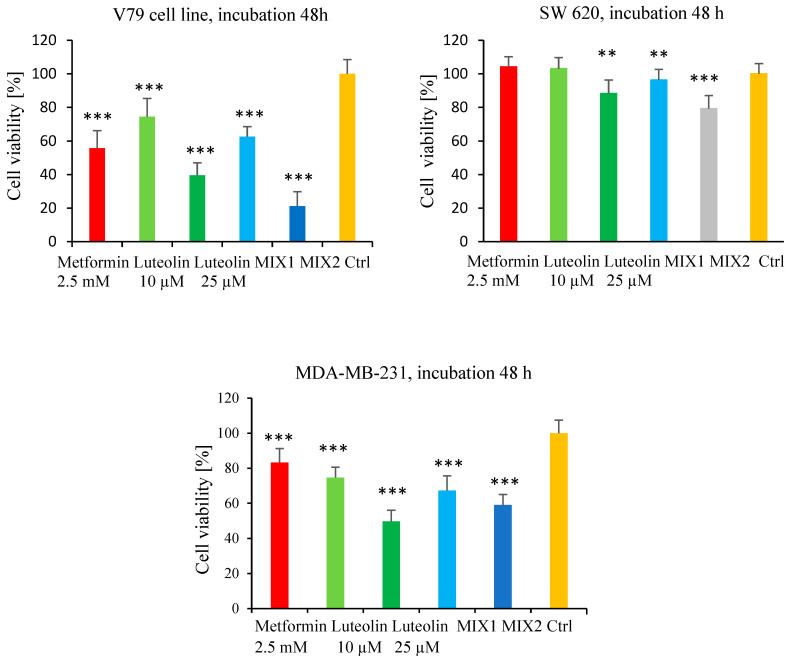
Effect of combined treatment with luteolin and metformin on the viability of MDA-MB-231, SW620, and V79 cells, assessed by the MTT assay. Mix 1: luteolin (10 μM) + metformin (2.5 mM); Mix 2: luteolin (25 μM) + metformin (2.5 mM). Absorbance values of treated cells (E) are expressed relative to untreated control cells (E_0_) after 48 h of incubation. Each bar represents the mean ± SD from three independent experiments. ** *p* < 0.01; *** *p* < 0.001.

In the MDA-MB-231 cell line (Figure 4), MIX 1 resulted in greater cytotoxicity compared with single treatments (83.28% for metformin and 74.63% for luteolin), leading to a statistically significant reduction in cell viability to 67.28%. The lowest viability (49.71%) was observed for luteolin at 25 µM. Application of MIX 2 did not further reduce viability compared with luteolin (25 µM) alone (59.10%).

In the SW620 cell line (Figure 4), a decrease in cell viability was observed after treatment with the mixtures compared with individual compounds, indicating an additive effect of metformin and luteolin. Treatment with metformin (2.5 mM) and luteolin (10 µM) alone slightly increased cell viability (104.19% and 103.14%, respectively) relative to the control (100%). However, MIX 1 (2.5 mM metformin + 10 µM luteolin) significantly reduced viability to 96.30%. A similar pattern was observed at the higher luteolin concentration: luteolin (25 µM) alone reduced viability to 88.34%, while MIX 2 (2.5 mM metformin + 25 µM luteolin) further decreased it to 79.25%, which was statistically significant.

Data for the normal V79 cell line are also presented in Figure 4. After 48 h of incubation with the tested compounds, a marked reduction in cell viability was observed compared with the control. Treatment with metformin (2.5 mM) resulted in 55.69% ± 10.5 viability, while luteolin at 10 µM and 25 µM reduced viability to 74.54% ± 10.8 and 39.63% ± 7.4, respectively.

For the combinations of both compounds, a further decrease in viability was recorded—for MIX 1 (metformin 2.5 mM + luteolin 10 µM), it was 62.64% ± 6.0, and, for MIX 2 (metformin 2.5 mM + luteolin 25 µM), 21.8% ± 8.6—representing a statistically significant reduction compared with the control (100% ± 8.4).

The obtained results indicate that the combined action of metformin and luteolin in V79 cells is additive, leading to a stronger cytotoxic effect compared with either compound used alone.

### 2.2. Effect of Luteolin and Metformin Under Oxidative Stress Conditions

After 48 h of incubation with metformin (2.5 mM), luteolin (10 µM and 25 µM), and their combinations, SW620, MDA-MB-231, and V79 cells were analyzed under two conditions: without stimulation (–H_2_O_2_) and after oxidative stress induction (+H_2_O_2_; 0.1 mM H_2_O_2_). In this study, the analysis focused on comparing intracellular reactive oxygen species (ROS) levels between –H_2_O_2_ and +H_2_O_2_ conditions for each treatment group.

**Figure 5:**
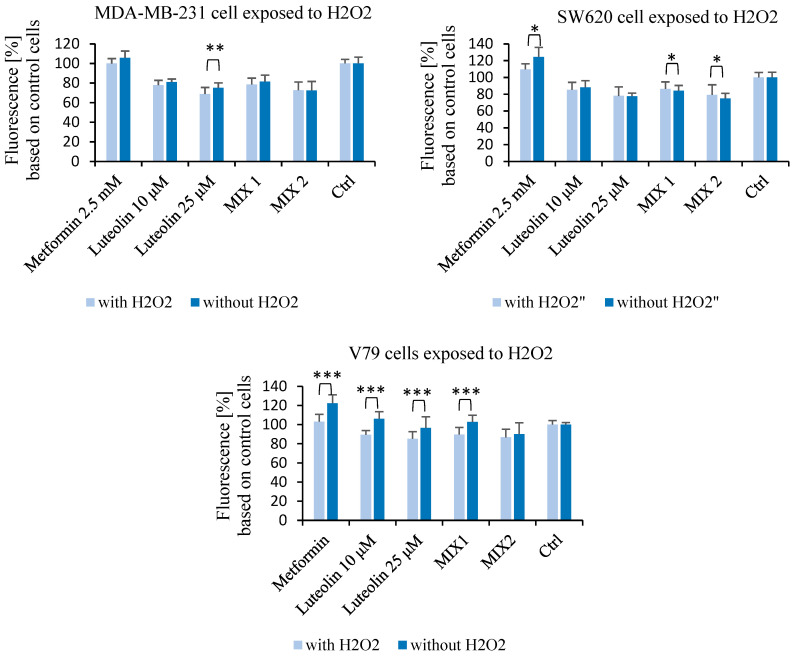
Intracellular reactive oxygen species (ROS) levels measured using the DCF-DA assay in MDA-MB-231, SW620, and V79 cells after 48 h of incubation with metformin (2.5 mM), luteolin (10 μM or 25 μM), and their combinations (Mix 1: metformin 2.5 mM + luteolin 10 μM; Mix 2: metformin 2.5 mM + luteolin 25 μM). Cells were analyzed under two conditions: without stimulation (−H_2_O_2_) and after oxidative stress induction (+H_2_O_2_; 0.1 mM). Data are presented as the mean ± SD from three independent experiments. Statistical significance refers to comparisons between −H_2_O_2_ and +H_2_O_2_ conditions within the same treatment group (* *p* ≤ 0.05; ** *p* < 0.01; *** *p* < 0.001).

In MDA-MB-231 cells, the ROS level after H_2_O_2_ stimulation differed significantly from the baseline in the group treated with luteolin at 25 µM. In SW620 cells treated with metformin (2.5 mM), a statistically significant increase in intracellular ROS levels was observed under non-stimulated conditions (–H_2_O_2_) compared with H_2_O_2_-induced cells. Furthermore, in SW620 cells, treatment with the combination of metformin and luteolin resulted in a statistically significant increase in ROS levels after H_2_O_2_ stimulation compared with the non-induced condition. In normal V79 cells, metformin (2.5 mM), luteolin (25 µM), and the mixture of metformin and luteolin (10 µM) led to a decrease in ROS levels after H_2_O_2_ stimulation compared with non-induced conditions (Figure 5).

### 2.3. Metabolomics Analysis

#### Identification of Intracellular and Extracellular Metabolites in V79, MDA-MB-231, and SW620 Cell Lines

A total of 37 intracellular metabolites were identified in V79 cells, 38 in SW620 cells, and 37 in MDA-MB-231 cells. In the extracellular fraction, 25 metabolites were commonly detected across all three cell lines. Cells were incubated for 1 h with luteolin (10 µM), metformin (2.5 mM), or their combination prior to extraction and subsequent ^1^H NMR analysis.

The chemical shift assignments for each metabolite are provided in the Supplementary Materials (Tables S1 and S2). Representative ^1^H NMR spectra of intracellular (Figures S1–S3) and extracellular (Figures S4–S6) metabolites are also included in the Supplementary Materials.

### 2.4. Multivariate Data Analysis

#### Intracellular and Extracellular Metabolism

Multivariate data analysis was performed to evaluate differences between intracellular and extracellular metabolite profiles. Principal Component Analysis (PCA), an unsupervised method, was applied to visualize global trends, identify clustering patterns, and detect potential outliers among the samples. Subsequently, a supervised Orthogonal Partial Least Squares Discriminant Analysis (OPLS-DA) was conducted to compare experimental groups (metformin, luteolin, and the combination of both) with the control.

Multivariate data analysis was performed to evaluate differences between intracellular and extracellular metabolite profiles. Principal Component Analysis (PCA), an unsupervised method, was applied to visualize global trends, identify clustering patterns, and detect potential outliers among the samples. Subsequently, a supervised Orthogonal Partial Least Squares Discriminant Analysis (OPLS-DA) was conducted to compare the experimental groups (metformin, luteolin, and their combination) with the control.

The OPLS-DA model parameters (R^2^X, R^2^Y, and Q^2^), along with the PCA results, are presented in the Supplementary Materials (intracellular metabolome: Figures S7–S9; extracellular metabolome: Figures S10–S12). The corresponding statistical parameters of the PCA and OPLS-DA models are summarized in Tables S3–S8.

For the V79 cell line, the OPLS-DA model revealed a clear statistical separation between the control and luteolin-treated samples (CV-ANOVA, *p* = 2.58 × 10^−7^) (Table S3 and Figure S7).

In contrast, no statistically significant group discrimination was observed for the SW620 and MDA-MB-231 cell lines in either the intracellular (Tables S4 and S5, Figures S8 and S9) or extracellular datasets (Figures S10–S12).

### 2.5. Metabolite Relative Concentrations and Statistical Analysis

#### 2.5.1. Intracellular Metabolites

**Figure 6:**
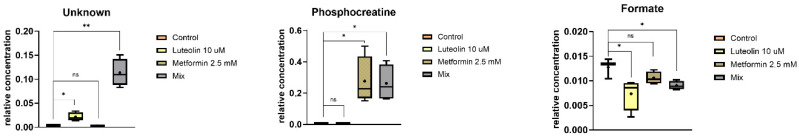
Box-and-whisker plots showing the relative concentrations of significantly altered intracellular metabolites in V-79 cells after incubation with different treatments. The boxes represent the interquartile range (IQR), encompassing the middle 50% of the data, while the whiskers indicate the minimum and maximum values. The median is shown as a horizontal line within each box, and the mean is marked with a cross (“+”). Statistical significance is denoted by asterisks (* *p* < 0.05; ** *p* < 0.01), while “ns” indicates non-significant differences.

**Figure 7:**
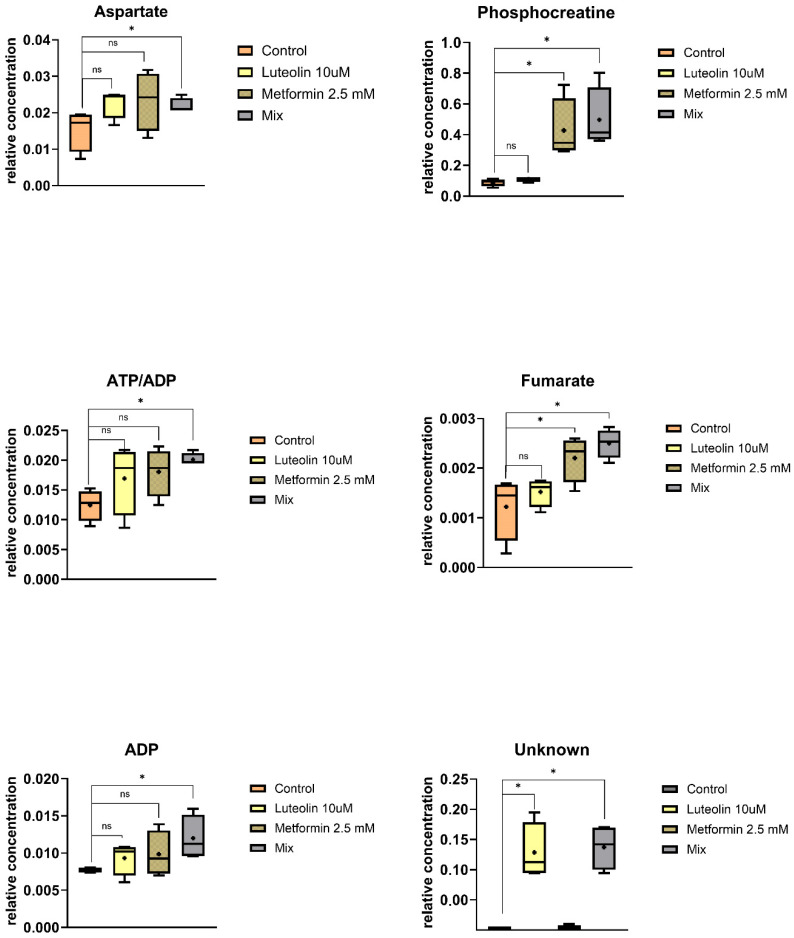
Box-and-whisker plots showing the relative concentrations of significantly altered intracellular metabolites in SW620 cells after incubation with different treatments. The boxes represent the interquartile range (IQR), encompassing the middle 50% of the data, while the whiskers indicate the minimum and maximum values. The median is shown as a horizontal line within each box, and the mean is marked with a cross (“+”). Statistical significance is denoted by asterisks (* *p* < 0.05), while “ns” indicates non-significant differences.

**Figure 8:**
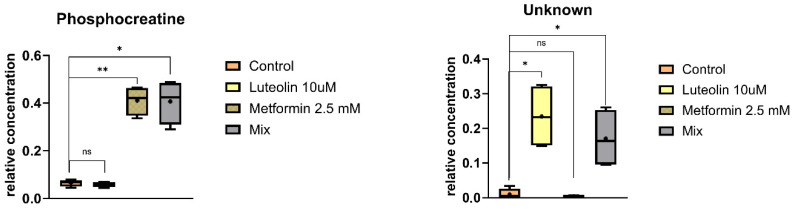
Box-and-whisker plots showing the relative concentrations of significantly altered intracellular metabolites in MDA-MB-231 cells after incubation with different treatments. The boxes represent the interquartile range (IQR), encompassing the middle 50% of the data, while the whiskers indicate the minimum and maximum values. The median is shown as a horizontal line within each box, and the mean is marked with a cross (“+”). Statistical significance is denoted by asterisks (* *p* < 0.05; ** *p* < 0.01), while “ns” indicates non-significant differences.

Table S9 (Supplementary Materials) summarizes the relative concentrations of intracellular metabolites in control samples and those incubated with luteolin, metformin, or their combination. Metabolomic differences between control and treated samples across the three cell lines (V79, SW620, and MDA-MB-231) are illustrated in Figure 6, Figure 7 and Figure 8.

In the V79 cell line, significant intracellular alterations were detected for an unidentified metabolite, phosphocreatine, and formate (Figure 6). Treatment with metformin resulted in an increase in phosphocreatine levels compared with the control. The combination of luteolin and metformin (Mix) caused a statistically significant elevation in both phosphocreatine and the unidentified metabolite, accompanied by a decrease in formate concentration. In contrast, luteolin alone decreased formate levels while increasing the unidentified metabolite (Figure 6).

In the SW620 cell line, the highest number of statistically significant intracellular alterations was observed (Figure 7). Incubation with metformin resulted in a marked and statistically significant increase in the relative concentrations of fumarate and phosphocreatine compared with the control. Combined treatment with luteolin and metformin (Mix) led to a significant elevation in phosphocreatine, aspartate, ADP, the overlapping ATP/ADP signal, and fumarate levels. Luteolin alone exerted a weaker effect, causing no statistically significant changes in most of the analyzed metabolites, except for the unidentified one (Figure 7).

The smallest metabolic alterations were observed in the MDA-MB-231 cell line, where a significant increase in phosphocreatine levels was detected following both metformin treatment alone and combined treatment with luteolin and metformin (Figure 8).

#### 2.5.2. Extracellular Metabolites

Table S10 presents a comparison of the relative concentrations of metabolites in control samples and those incubated with the tested compounds (Supplementary Materials).

**Figure 9:**
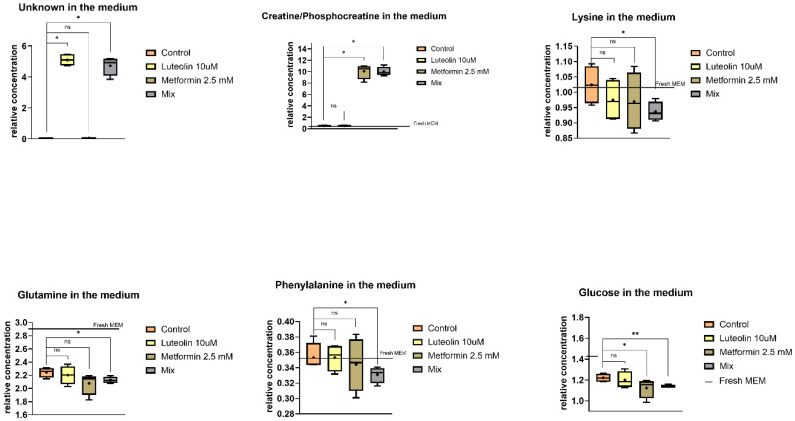
Box-and-whisker plots showing the relative concentrations of significantly altered extracellular metabolites in V-79 cells after incubation with different treatments. The boxes represent the interquartile range (IQR), encompassing the middle 50% of the data, while the whiskers indicate the minimum and maximum values. The median is shown as a horizontal line within each box, and the mean is marked with a cross (“+”). Statistical significance is denoted by asterisks (* *p* < 0.05; ** *p* < 0.01), while “ns” indicates non-significant differences. Metabolite abundances are shown for control samples and for cells treated with various compounds, relative to levels in fresh MEM medium.

**Figure 10:**
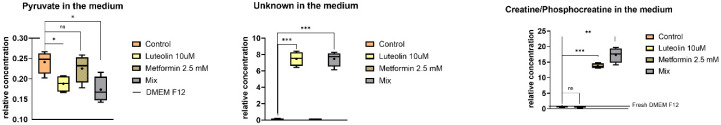
Box-and-whisker plots showing the relative concentrations of significantly altered extracellular metabolites in SW620 cells after incubation with different treatments. The boxes represent the interquartile range (IQR), encompassing the middle 50% of the data, while the whiskers indicate the minimum and maximum values. The median is shown as a horizontal line within each box, and the mean is marked with a cross (“+”). Statistical significance is denoted by asterisks (* *p* < 0.05; ** *p* < 0.01; *** *p* < 0.001), while “ns” indicates non-significant differences. Metabolite abundances are shown for control samples and for cells treated with various compounds, relative to levels in fresh DMEM/F-12 medium.

**Figure 11:**
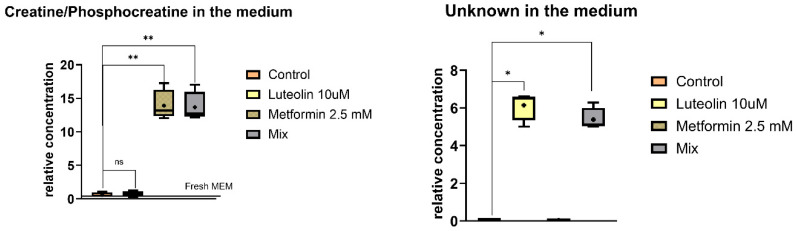
Box-and-whisker plots showing the relative concentrations of significantly altered extracellular metabolites in MDA-MB-231 cells after incubation with different treatments. The boxes represent the interquartile range (IQR), encompassing the middle 50% of the data, while the whiskers indicate the minimum and maximum values. The median is shown as a horizontal line within each box, and the mean is marked with a cross (“+”). Statistical significance is denoted by asterisks (* *p* < 0.05; ** *p* < 0.01), while “ns” indicates non-significant differences. Metabolite abundances are shown for control samples and for cells treated with various compounds, relative to levels in fresh MEM medium.

The metabolomic differences between the control and the samples treated with luteolin, metformin, or their combination are illustrated in Figure 9, Figure 10 and Figure 11. In the extracellular medium of V79 cells, significant alterations were detected in several metabolites (Figure 9).

Treatment with luteolin resulted in a significant increase in only one metabolite—an unidentified compound. In contrast, metformin treatment led to elevated levels of creatine/phosphocreatine and a decrease in glucose concentration in the medium.

The combination of luteolin and metformin caused a pronounced increase in creatine/phosphocreatine and the unidentified metabolite, accompanied by a decrease in phenylalanine, lysine, glutamine, and glucose levels (Figure 9). Luteolin alone exhibited a weaker effect, whereas its combination with metformin markedly enhanced the overall metabolic response, suggesting an additive influence of both compounds on cellular energy and amino acid metabolism. Luteolin alone exhibited a weaker effect, whereas its combination with metformin markedly enhanced the overall metabolic response, suggesting a synergistic impact of both compounds on cellular energy and amino acid metabolism.

In the culture medium of SW620 cells, significant alterations were observed in several extracellular metabolite levels (Figure 10). Metformin treatment resulted in a significant increase in creatine/phosphocreatine levels compared with the control.

The combination of luteolin and metformin caused a pronounced increase in both creatine/phosphocreatine and the unidentified metabolite, accompanied by a decrease in pyruvate concentration in the medium. Treatment with luteolin alone led to a significant increase in the unidentified compound and a decrease in pyruvate levels.

The smallest alterations in extracellular metabolite levels were observed in the MDA-MB-231 cell line (Figure 11). Following metformin treatment, an increase in creatine/phosphocreatine concentration was detected in the culture medium. In the case of the combined treatment with luteolin and metformin, elevated levels of both the unidentified metabolite and creatine/phosphocreatine were observed.

### 2.6. Pathway Analysis

To investigate the metabolomic effects of luteolin, metformin, and their combination on three cell lines (MDA-MB-231, SW620, and V79), Metabolite Set Enrichment Analysis (MSEA) and Pathway Analysis (PA) were performed using MetaboAnalyst 5.0 (https://www.metaboanalyst.ca/). MSEA was used to identify metabolite sets associated with specific cellular phenotypes, with pathways considered significant if both the Holm-adjusted *p*-value and the FDR-adjusted *p*-value were <0.05, and the HITS value exceeded 1.

PA was applied to determine pathway associations using the same significance criteria as for MSEA. Due to the limited number of statistically significant intracellular metabolites detected in the V79 and MDA-MB-231 cell lines, MSEA and PA analyses were conducted exclusively for the SW620 cell line incubated with the combination (MIX) of luteolin and metformin.

**Figure 12:**
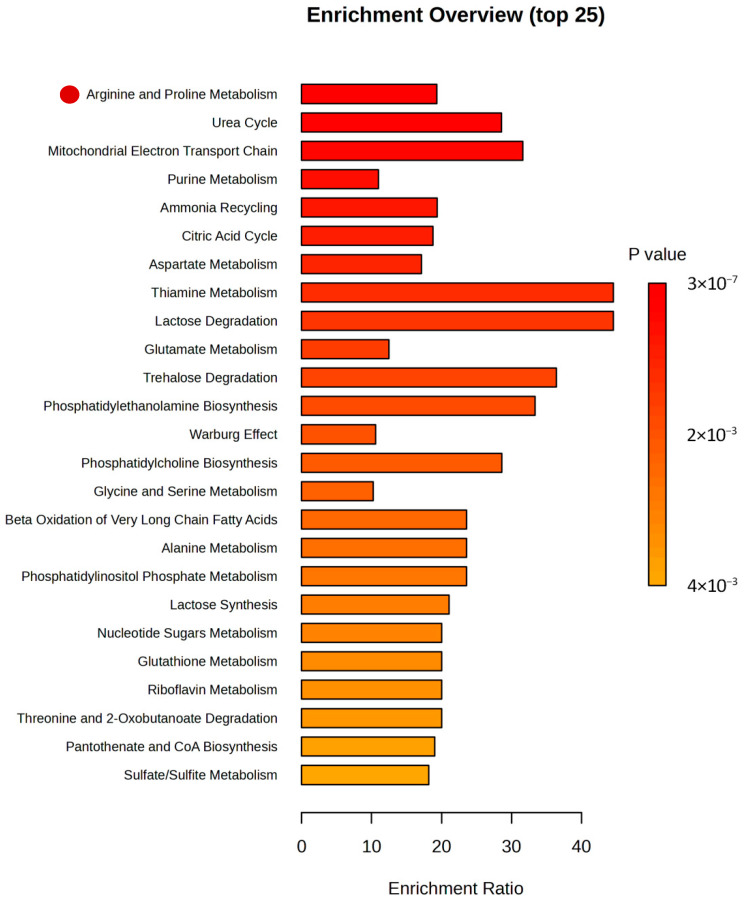
Summary plot of significantly enriched metabolic pathways identified after treatment of SW620 cells with luteolin (10 µM) and metformin (2.5 mM). Pathway enrichment analysis was performed using MetaboAnalyst 5.0, based on Holm-adjusted *p*-values, raw *p*-values, and false discovery rate (FDR). Statistically significant intracellular metabolites were analyzed to identify the most enriched pathways. Red dots indicate metabolic pathways that meet the significance criteria in both MSEA and PA analyses.

The enriched pathways are presented in Figure 12. Based on the MSEA results, 25 enrichment pathways met the defined significance criteria. Among them, the following pathways were found to fulfill all required thresholds: Arginine and Proline Metabolism, Urea Cycle, Mitochondrial Electron Transport Chain, Purine Metabolism, Ammonia Recycling, Citric Acid Cycle, and Aspartate Metabolism. Additional parameters used for these analyses are summarized in Table 1. In contrast, according to the results of the Pathway Analysis (PA), only the Arginine Biosynthesis pathway satisfied the required criteria (Table 2).

## 3. Discussion

Metformin is a well-established and effective drug used in the treatment of patients with type 2 diabetes (T2DM). However, an increased risk of cancer has been observed in diabetic patients. Risk factors for the development of both diabetes and cancer include age, sex, obesity, reduced physical activity, and poor dietary habits [2]. Numerous epidemiological studies have demonstrated a link between metformin use and the prevention and treatment of cancer. Therefore, our study aimed to investigate the additive anticancer effects of metformin and the natural polyphenol luteolin.

Luteolin exhibits anticancer activity in colorectal cancer by inhibiting the Wnt/IGF signaling pathway while simultaneously promoting natural anti-oncogenic mechanisms, such as the pro-apoptotic pathway mediated by the expression of BAX proteins, caspase-3, -7, and -9, and the tissue inhibitor of metalloproteinases (TIMP). Luteolin inhibits matrix metalloproteinases (MMPs), which are involved in the degradation and remodeling of the extracellular matrix (ECM)—processes that promote tumor progression. It also induces antioxidant enzymes such as superoxide dismutase (SOD), thereby reducing the levels of reactive oxygen species. Moreover, luteolin inhibits hyperglycemia and reduces proinflammatory cytokines, both of which are linked to tumor proliferation and progression [16].

Similarly, metformin may act on proinflammatory cytokines present in the tumor microenvironment, inhibiting the growth of susceptible tumors such as breast cancer [41]. In our study, a dose-dependent decrease in the viability of MDA-MB-231 breast cancer cells and V79 hamster fibroblasts was observed at both time points following luteolin treatment. The cytotoxic effect of metformin on MDA-MB-231 cells was evident only after extended incubation for 48 h. Paula Cabello et al. investigated the effects of metformin at concentrations ranging from 1 mM to 40 mM on the viability of MDA-MB-231 cells after 24, 48, and 72 h of incubation. The study demonstrated that metformin at concentrations of 10 mM and higher significantly reduced cell viability after 48 and 72 h, whereas, at 24 h, significant inhibition was observed only at 40 mM [37].

Previous studies have also reported antiproliferative and antimetastatic effects of luteolin against androgen receptor (AR)-positive triple-negative breast cancer (TNBC) cell lines. Luteolin significantly inhibited the proliferation and metastasis of AR-positive TNBC cells. Furthermore, it inactivated the AKT/mTOR signaling pathway and reversed epithelial–mesenchymal transition (EMT). The combination of luteolin with AKT/mTOR inhibitors synergistically inhibited the proliferation and metastasis of AR-positive TNBC cells [33].

The results of Han-Tsang Wu et al. support our findings, showing a dose-dependent decrease in the viability of breast cancer cells treated with luteolin. They observed that luteolin arrested the cell cycle in the S phase, reduced telomerase levels, and inhibited the phosphorylation of the NF-κB α inhibitor in MDA-MB-231 cells. Other researchers have suggested that monoacetylated derivatives of luteolin possess stronger antiproliferative and antioxidant activity against MDA-MB-231 breast cancer cells and HCT116 colorectal cancer cells compared with luteolin alone [42,43,44]. Studies by Kang et al. demonstrated that luteolin induces apoptosis in colorectal cancer cells through activation of the nuclear factor erythroid 2-related factor 2 (Nrf2), triggered by promoter DNA demethylation, and increases the expression of pro-apoptotic proteins and antioxidant enzymes [39].

In our study, metformin did not exhibit cytotoxic effects on SW620 colorectal cancer cells. This observation is consistent with the findings of X. Sui et al., who reported that metformin did not inhibit proliferation or induce apoptosis in three colorectal cancer cell lines at concentrations of 1, 5, and 10 mmol/L [45]. Most studies indicate that metformin reduces the risk of colorectal cancer and improves prognosis. Interestingly, Mogavero et al. found that metformin inhibited proliferation and migration by inducing cell cycle arrest at the G0/G1 phase, reducing c-Myc expression, and downregulating IGF1R in three CRC cell lines. However, metformin did not induce apoptosis, autophagy, or senescence in HT29, HCT116, and HCT116 p53−/− cells, and it only transiently inhibited CRC cell proliferation [38].

Selected randomized studies have suggested that metformin may be associated with a significant reduction in cancer incidence and mortality in colorectal, pancreatic, gastric, and esophageal cancers. However, in the same studies, no association was found between metformin use and the risk of breast, prostate, lung, or ovarian cancer [46].

In our study, it was demonstrated that simultaneous incubation with a mixture of luteolin and metformin significantly inhibited the viability of SW620 and MDA-MB-231 cell lines compared with monotherapy. An enhanced cytotoxic effect was observed following the combined use of both compounds, and this effect was also evident in V79 cells.

Metformin has been investigated in combination with various agents, such as 5-fluorouracil (5-FU), a first-line drug for advanced colorectal cancer (CRC) therapy. It has been shown that pretreatment with metformin followed by 5-FU significantly inhibited proliferation and both early and late apoptosis in SW620 cells compared with 5-FU monotherapy [47]. The combination of metformin and 5-FU induced cell cycle arrest in 5-FU-resistant CRC cells, as reported by Kim et al. [48]. The synergistic effect of metformin and oxaliplatin was evident through enhanced apoptosis and inhibition of proliferation and migration of chemoresistant HT-29 and HCT-116 cells compared with individual drug treatments [49]. Increased caspase-3 expression and other pro-apoptotic factors were reported in a study on the combination of metformin and silibinin, a flavonoid used as a dietary supplement in liver diseases [50]. A study on the combined use of metformin and 5-aminosalicylic acid (5-ASA, mesalazine), a drug commonly used to treat ulcerative colitis (UC) and Crohn’s disease (CD), demonstrated pro-apoptotic properties through increased oxidative stress in cancer cells [8].

A clinical study conducted in the United States among diabetic patients treated with metformin showed a significantly higher pathological complete response (pCR) rate after neoadjuvant chemotherapy for invasive breast cancer compared with patients not treated with metformin or without diabetes [3]. In contrast, research by Soley Bayraktar indicated that metformin use during adjuvant chemotherapy had no significant impact on survival in patients with non-triple-negative breast cancer (TNBC) [51]. In the study by Yang Yan et al., conducted on a rat model of liver injury induced by carbon tetrachloride (CCl_4_), the combination of metformin and luteolin showed a synergistic effect and improved liver function. Increased expression of antioxidant enzymes, decreased levels of inflammatory cytokines (IL-6, TNF-α, IL-1β), and reduced apoptosis via downregulation of apoptotic proteins (caspase-3, BAX) and upregulation of anti-apoptotic proteins (BCL-2) were observed [52].

Reactive oxygen species (ROS) cause oxidative stress (OS), which can damage multiple cellular components involved in maintaining redox homeostasis and intracellular signaling pathways. Disruption of this balance initiates numerous pathological processes and contributes to cancer development. The observed and expected increase in the antioxidant activity of the metformin–luteolin combination can be explained by their complementary mechanisms of action.

Metformin indirectly reduces oxidative stress by activating AMPK and inhibiting mitochondrial complex I, leading to decreased ROS generation and improved redox homeostasis. Luteolin, in turn, acts as a direct ROS scavenger and enhances the antioxidant defense system through Nrf2 activation and upregulation of related enzymes such as SOD and catalase. Therefore, their combined use was expected to potentiate antioxidant effects by targeting both mitochondrial and cytoplasmic sources of oxidative stress, as previously reported for similar polyphenol–metformin combinations [34,35,36].

In our study, metformin significantly increased ROS levels in SW620 cells (both with and without H_2_O_2_ stimulation), while a weaker effect was observed in MDA-MB-231 cells. Treatment with luteolin and the combined use of both compounds decreased ROS levels in all three cell lines. In MDA-MB-231 cells under induced oxidative stress, luteolin at 25 µM reduced ROS levels, whereas in SW620 cells, the combination of luteolin and metformin significantly increased ROS compared with non-induced cells.

ROS plays a complex role in cancer, as it may promote either cell survival or apoptosis depending on its concentration and the type of cancer cell [53]. In the study by Mogavero et al., metformin increased ROS levels in HCT116 and HCT116 p53−/− cells (to a lesser extent in HT29) and induced mitochondrial depolarization. It may thus act by increasing ROS levels and inhibiting mTOR [38,54]. Metformin has also been shown to sensitize MCF-7 cells to radiation, but not MDA-MB-231 cells, by inducing ROS production and AMPK activation in luminal but not basal breast cancer cells [47]. In the study by Chang et al., the effect of luteolin was examined on non-cancerous H9C2 cardiomyocytes treated with 100 µM H_2_O_2_. It was shown that ROS generation was significantly reduced by luteolin treatment [55]. Similar observations were made by Kuang et al., who studied the effect of metformin on human non-cancerous periodontal ligament cells (hPDLCs) treated with H_2_O_2_. Within 2 h after H_2_O_2_ addition, ROS levels significantly increased, whereas pre-incubation with 100 µM metformin reduced ROS accumulation [56].

The dual role of reactive oxygen species (ROS) in cancer cell physiology should be considered when interpreting the observed effects. Moderate ROS levels are often associated with adaptive stress responses that support tumor cell survival, proliferation, and resistance to therapy. However, excessive ROS accumulation can disrupt redox homeostasis and trigger apoptotic pathways.

In the present study, the combination of metformin and luteolin increased ROS levels in SW620 cells compared with individual treatments. This effect may reflect a shift from physiological to cytotoxic oxidative stress. Metformin inhibits mitochondrial complex I, leading to impaired electron transport and increased mitochondrial ROS generation, while luteolin may additionally interfere with antioxidant defense mechanisms such as Nrf2 signaling. The concurrent action of both compounds could therefore intensify oxidative imbalance, contributing to mitochondrial dysfunction and apoptosis rather than promoting stress tolerance. This interpretation is consistent with previous findings showing that combined pro-oxidant and metabolic stress can enhance apoptosis in colorectal cancer cells [36,37,40].

To better understand the metabolic responses underlying the observed biological effects, an NMR-based metabolomic analysis was performed on three cell lines—SW620, MDA-MB-231, and V79—following short-term (1 h) incubation with luteolin (10 µM), metformin (2.5 mM), and their combination. The selected concentrations were non-cytotoxic or only mildly cytotoxic but remained biologically active, as confirmed by MTT assay results. This approach enabled the detection of early metabolic alterations occurring before major effects on cell viability. The one-hour incubation period allowed observation of rapid metabolic shifts directly associated with the compounds’ interaction with central energy and redox pathways, rather than secondary effects resulting from prolonged exposure. Both intracellular and extracellular metabolites were analyzed to provide a comprehensive overview of how the treatments influenced energy metabolism, amino acid turnover, and redox balance.

Short-term (1 h) exposure of V79 cells to luteolin (10 µM), metformin (2.5 mM), and their combination revealed distinct intra- and extracellular metabolic alterations. Intracellularly, significant changes were observed in an unidentified metabolite, phosphocreatine, and formate, suggesting modulation of energy metabolism and one-carbon flux.

Metformin increased phosphocreatine levels, which may indicate a temporary activation of the creatine–phosphocreatine energy buffer. This rise in phosphocreatine could also reflect the activation of protective Cr/CK and mitochondrial-stabilizing mechanisms, as phosphocreatine has been shown to protect mitochondria by reducing membrane permeability, limiting ROS production, and preventing apoptosis through both energy-dependent and -independent pathways [57].

Luteolin alone decreased formate and increased the unidentified metabolite, whereas the combination further elevated phosphocreatine and the unidentified metabolite while maintaining lower formate levels. A decrease in formate may reflect suppression of folate-linked one-carbon flux and aligns with the “formate-driven metabolic switch” connecting nucleotide and energy metabolism [58]. These observations suggest, but do not confirm, the involvement of folate-dependent one-carbon and redox pathways.

Metformin increased the extracellular levels of creatine/phosphocreatine (overlapping signal) and decreased glucose, consistent with enhanced glucose utilization and AMPK-linked energy responses previously described for metformin [59]. Luteolin alone elevated only the unidentified metabolite, while the combination produced additive effects, resulting in higher creatine/phosphocreatine and lower phenylalanine, lysine, glutamine, and glucose levels. Overall, these findings suggest that luteolin and metformin may jointly modulate energy metabolism, amino acid turnover, and one-carbon fluxes in V79 cells, indicating an early adaptive response aimed at maintaining energy and redox balance.

The metabolism of cancer cells shifts toward aerobic glycolysis, regardless of oxygen availability, leading to increased nutrient uptake and significant lactate accumulation. This phenomenon, known as the Warburg effect, was first described by Otto Warburg in 1924. Oncogenes such as Myc, nuclear factor kappa B (NF-κB), and tyrosine kinase growth factor receptors—including the insulin-like growth factor-1 receptor (IGF1R) and the epidermal growth factor receptor (HER2)—activate proliferative pathways such as phosphoinositide 3-kinase (PI3K) and the mammalian target of rapamycin (mTOR), resulting in the transcription of genes involved in glycolysis.

In contrast, AMP-activated protein kinase (AMPK), activated by metformin, acts as a suppressor, inhibiting proliferative metabolism under conditions of oxidative stress and energy deficiency. AMPK interferes with the expression of genes involved in gluconeogenesis, lipogenesis, protein synthesis, and potentially angiogenesis [3]. Metabolic profiling of cancer can enable early diagnosis, monitoring of treatment response, and the identification of biomarkers and novel therapeutic targets. In our study, NMR-based metabolomic analysis revealed differences in both intracellular and extracellular metabolite profiles following treatment with metformin, luteolin, and their combination. Metabolite Set Enrichment Analysis (MSEA) and pathway analysis were performed only for metabolites obtained from SW620 cells incubated with the combination of metformin and luteolin. Analysis was not conducted for the other cell lines due to the limited number of statistically significant metabolites.

Creatine phosphate was detected in both intracellular and extracellular fractions, and its levels significantly increased following metformin and combination treatment. Metformin may lead to lactic acidosis as a result of intensified anaerobic glucose metabolism [60]. Creatinine is also produced under anaerobic conditions, where the phosphate group from phosphocreatine is transferred to ADP, resulting in creatinine formation [61]. The presence of phosphocreatine in treated samples may therefore reflect enhanced anaerobic metabolism.

Jain et al. reported a correlation between the cell proliferation rate (including in breast cancer) and increased glycine metabolism [62]. Xie et al. observed that breast cancer was associated with lower levels of circulating aspartate in plasma and tumor tissue compared with adjacent non-tumorous tissue, likely due to increased aspartate utilization by tumor cells [63].

In our intracellular metabolite analysis, elevated levels of aspartate, fumarate, ATP/ADP*, and ADP were observed in SW620 cells treated with metformin alone or in combination with luteolin. This may indicate enhanced tricarboxylic acid (TCA) cycle activity, which, in addition to citrate, provides precursors for amino acid biosynthesis [64].

Vincent Richard et al. reported significant alterations in lactate, pyruvate, glutamine, glucose, alanine, and acetate in breast cancer cells, with an inverse correlation between lactate levels and tumor size—lower plasma lactate levels were associated with larger tumors [65]. In our study, extracellular pyruvate levels decreased in SW620 cells following treatment with luteolin and the luteolin–metformin combination, suggesting a cellular shift toward alternative energy sources, such as glycolysis and amino acid metabolism [66].

It should be emphasized that the incubation period in this study was limited to 1 h, allowing the detection of early metabolic responses rather than long-term antiproliferative or cytotoxic effects. The observed alterations therefore likely reflect rapid adaptive shifts in energy and redox metabolism, including enhanced TCA cycle activity and activation of the creatine–phosphocreatine buffering system.

In contrast, the MDA-MB-231 cell line exhibited only minor metabolic changes after short-term treatment, mainly limited to increased phosphocreatine and creatine levels following metformin or combined treatment. This modest response may be attributed to the pronounced glycolytic phenotype and metabolic flexibility of triple-negative breast cancer cells, which may require longer incubation to exhibit more extensive metabolic reprogramming. Together, these findings indicate that metformin and luteolin induce early, cell type–dependent metabolic adaptations that precede potential antiproliferative outcomes.

Overall, the results obtained from the three cellular models suggest a consistent yet cell type–specific pattern of metabolic modulation. In non-tumorigenic V79 fibroblasts, both compounds primarily triggered transient metabolic adjustments related to energy buffering and one-carbon metabolism, indicative of early adaptive and cytoprotective responses rather than cytotoxicity.

In contrast, SW620 colorectal cancer cells exhibited the most pronounced alterations, particularly under combined treatment with metformin and luteolin, where enhanced TCA cycle activity, increased creatine–phosphocreatine turnover, and reduced extracellular pyruvate levels indicate intensified metabolic reprogramming consistent with antiproliferative activity.

The triple-negative breast cancer cell line MDA-MB-231 showed only modest metabolic shifts after 1 h of exposure, likely reflecting its high glycolytic capacity and metabolic plasticity. Longer incubation periods may be necessary to uncover downstream effects and confirm sustained reprogramming of energy and redox pathways in this more aggressive phenotype.

Together, these findings highlight that the additive effects of metformin and luteolin depend on the metabolic background of the cell type, with the strongest and most immediate response observed in SW620 cells. The observed metabolic alterations—including changes in TCA cycle intermediates, phosphocreatine buffering, and decreased extracellular pyruvate levels—are consistent with early AMPK-related metabolic regulation and potential suppression of anabolic mTOR signaling. These pathways are recognized as central regulators of tumor cell growth and survival under metabolic stress [67,68].

Therefore, the combined use of metformin and luteolin may exert synergistic effects by simultaneously modulating metabolic and signaling networks, enhancing redox balance, and reducing the biosynthetic capacity of cancer cells.

Although the NMR analysis focused on short-term (1 h) metabolic responses, the subsequent decrease in cell viability observed after 24–48 h supports the notion that these early metabolic shifts may initiate signaling cascades leading to growth inhibition. Thus, the metabolomic results complement the viability data and provide mechanistic insight into the time-dependent effects of metformin and luteolin.

## 4. Materials and Methods

### 4.1. Cell Culture

**Figure 13:**
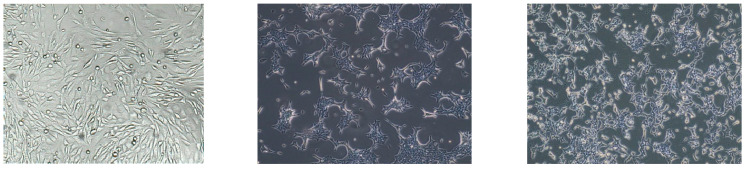
Representative microscopic images of MDA-MB-231, SW620, and V79 cell lines captured using the Invitrogen EVOS XL imaging system (Thermo Fisher Scientific, Massachusetts, United States) (magnification 200×).

Human breast adenocarcinoma cells (MDA-MB-231), human colorectal adenocarcinoma cells (SW620), and normal Chinese hamster lung fibroblasts (V79) were cultured in growth media appropriate for each cell line. MDA-MB-231 and V79 cells were maintained in EMEM (Minimum Essential Medium Eagle without L-glutamine), while SW620 cells were cultured in Dulbecco’s Modified Eagle Medium/Nutrient Mixture F-12 (DMEM/F12). All media were supplemented with 10% fetal bovine serum (FBS; Capricorn Scientific, Ebsdorfergrund, Germany) and supplemented with 10% FBS and 100 U/mL penicillin, 100 µg/mL streptomycin. Cells were incubated at 37 °C in a humidified atmosphere containing 5% CO_2_. Cultures were maintained in 75 cm^2^ flasks, with media replaced twice weekly or cells subcultured as required, depending on the proliferation rate and confluence. Representative images of MDA-MB-231 (breast cancer), SW620 (colorectal cancer), and V79 (normal fibroblast) cultures are presented in Figure 13.

### 4.2. Assessment of Cytotoxicity and Reactive Oxygen Species Levels

#### 4.2.1. Cell Viability Assay—MTT Test

The MTT assay is a sensitive colorimetric method used to evaluate cell viability based on metabolic activity. The principle of this method relies on the reduction of MTT (3-(4,5-dimethylthiazol-2-yl)-2,5-diphenyltetrazolium bromide) by mitochondrial succinate dehydrogenase to insoluble purple formazan crystals, which are subsequently solubilized in organic solvents.

MDA-MB-231, SW620, and V79 cells were seeded at a density of 1 × 10^4^ cells per well in sterile 96-well plates (TPP, Trasadingen, Switzerland). At the time of assay initiation, the cells were in the logarithmic growth phase. The cultures were incubated under standard conditions (5% CO_2_, 95% humidity, 37 °C) for 24 h prior to treatment. Following incubation, cells were exposed to metformin at concentrations of 1, 2.5, 5, 10, and 25 mM and to luteolin at concentrations of 1, 10, 25, 50, and 100 µM. To evaluate the potential additive effects of metformin and luteolin on the metabolic activity of the tested cell lines, cells were also treated with combinations of metformin (2.5 mM) and luteolin (10 µM or 25 µM).

The concentrations of metformin and luteolin used in this study were selected based on preliminary cytotoxicity tests and literature data describing the most common concentration ranges applied in in vitro models. Concentrations of 1–25 mM for metformin and 1–100 µM for luteolin were chosen to represent low, moderate, and high activity levels, allowing comparative analysis of dose-dependent effects. Cell viability was assessed at two time points—24 h and 48 h. After incubation, the treatment solutions were removed, and cells were washed with phosphate-buffered saline (PBS). The cells were then incubated with MTT solution (BioReagent, >97.5%, Sigma-Aldrich, Gillingham, UK) for 2 h. Subsequently, the MTT solution was removed, and the resulting formazan crystals were dissolved in isopropanol (≥99.7%, PolAura, Olsztyn, Poland). Absorbance was measured using a Varioskan microplate reader (PerkinElmer, Waltham, MA, USA) at a wavelength of 570 nm. The number of viable cells was directly proportional to the color intensity. Untreated cells served as the control. Each experiment was performed in three independent replicates. The percentage of viable cells was calculated according to the following equation:

$$Cell\;viability\;(\%)=\frac{sample\;absorbance}{control\;absorbance}\;\times \;100\%$$

#### 4.2.2. Measurement of Reactive Oxygen Species—DCF-DA Assay

The DCF-DA assay was used to measure intracellular reactive oxygen species (ROS) levels. DCFH_2_-DA (2′,7′-dichlorodihydrofluorescein diacetate) is a cell-permeable compound that diffuses across the plasma membrane and is subsequently deacetylated by intracellular esterases into non-fluorescent DCFH_2_ (2′,7′-dichlorodihydrofluorescein). This compound accumulates in the cytosol, where it is oxidized by reactive oxygen species to form the fluorescent product DCF (2′,7′-dichlorofluorescein). Because DCFH_2_ reacts with various types of ROS, the fluorescence intensity of DCF is directly proportional to the intracellular ROS level and the degree of oxidative stress. To induce oxidative stress, cells were exposed to 0.1 mM H_2_O_2_ for 1 h at 37 °C in a humidified 5% CO_2_ atmosphere. Following stimulation with hydrogen peroxide (H_2_O_2_), which induced oxidative stress in MDA-MB-231, SW620, and V79 cells, the cultures were treated with metformin (2.5 mM), luteolin (10 µM or 25 µM), or their combinations (2.5 mM metformin + 10 µM or 25 µM luteolin). After 48 h of incubation, the treatment solutions were removed, and the cells were rinsed with phosphate-buffered saline (PBS). They were then incubated with DCF-DA solution at a final concentration of 25 µM for 1 h in the dark. Fluorescence intensity was measured using a Varioskan microplate reader (PerkinElmer, Waltham, MA, USA) at excitation 485 nm and emission 520 nm.

Results were expressed as the E/E_0_ ratio, where *E* represents the fluorescence value of the treated group and *E_0_* corresponds to the untreated control. ROS levels were expressed as the percentage of DCF fluorescence intensity relative to the control. For ROS analysis, statistical comparisons were made between non-stimulated (without H_2_O_2_) and H_2_O_2_-induced (with H_2_O_2_) conditions within each treatment group.

#### 4.2.3. Statistical Analysis of Biological Activity Data

All experimental results were subjected to statistical analysis. The Shapiro–Wilk test was used to assess the normality of data distribution. For normally distributed data, statistical significance was evaluated using the Student’s *t*-test for independent samples. For non-normally distributed data, the Mann–Whitney *U* test was applied.

All results were compared to the control group. A *p*-value ≤ 0.05 was considered statistically significant. Statistical analyses were performed using STATISTICA, version 13.1 (TIBCO Software Inc., Palo Alto, CA, USA).

### 4.3. NMR Analysis

#### 4.3.1. Sample Preparation and ^1^H NMR Measurement

For metabolomic analysis, cells were cultured in 75 cm^2^ flasks until reaching confluence: 1.6 × 10^7^ for V79, 1.0 × 10^7^ for MDA-MB-231, and 8.0 × 10^6^ for SW620. After incubation with luteolin, metformin, or their combination at the selected concentrations (luteolin: 10 µM; metformin: 2.5 mM; combination: 10 µM luteolin + 2.5 mM metformin) for 1 h, the culture medium was collected, and the cells were washed with phosphate-buffered saline (PBS). Extracellular metabolites were collected from the culture medium, while the cells were gently rinsed with PBS to remove residual treatment solutions. Subsequently, the cells were placed on ice, and 2.5 mL of cold methanol/water (3:1, *v*/*v*) was added twice. The resulting cell suspensions were stored at −80 °C for further analysis.

#### 4.3.2. Sonication and Evaporation of Samples

The metabolomic analysis included four replicates for each treatment, both for intracellular and extracellular samples. Samples containing cells and methanol (3:1, *v*/*v*) were sonicated for 10 min to disrupt the cells and release intracellular metabolites. Subsequently, the samples were centrifuged at 4 °C and 14,000 rpm for 12 min. After centrifugation, the supernatant was collected and evaporated using a SpeedVac concentrator plus (Eppendorf, Hamburg, Deutschland) at 500 rpm and 45 °C. Extracellular samples were not subjected to sonication. Instead, methanol/water (3:1, *v*/*v*) was added, and the mixtures were homogenized for 10 min at 30 Hz with a metal bead, followed by centrifugation for 15 min at 10,000 rpm. After evaporation, 600 µL of the appropriate buffer was added: 0.1 M sodium phosphate buffer (pH 7.4) prepared in 50% D_2_O and containing 0.3 mM TSP (trimethylsilyl propionate) for cell extracts, and 0.4 M sodium phosphate buffer (pH 7.4) prepared in 100% D_2_O and containing 0.06 mM TSP for culture media.

Finally, 550 µL of each prepared sample was transferred to 5 mm NMR tubes (Norell, Morganton, NC, USA) for ^1^H NMR analysis.

#### 4.3.3. ^1^H NMR Spectroscopy Analysis

^1^H NMR spectra were acquired at an operating frequency of 600.58 MHz using a Bruker UltraShield™ Plus AV2 spectrometer (Bruker GmbH, Bremen, Germany). The cpmgpr1d pulse sequence was applied. The acquisition parameters were as follows: relaxation delay (RD) = 3.5 s, acquisition time = 2.75 s, number of scans = 128, mixing time = 125 ms, and time domain data points = 64 K.

Spectra were manually phased and processed using TopSpin 3.2 software (Bruker GmbH, Bremen, Germany). Baseline correction was performed using the Whittaker smoother algorithm in MestReNova v11.0.3 (Mestrelab Research, Qingdao, China). All spectra were referenced to the TSP resonance at 0.00 ppm.

#### 4.3.4. Univariate Processing and Statistical Data Analysis of 1D NMR Spectra

All spectra were processed using MATLAB R2014a (Natick, MA, USA). The water resonance region (4.508–5.051 ppm) was excluded for intracellular spectra, and the region (4.679–5.067 ppm) was excluded for extracellular spectra. Baseline and phase corrections were applied, and chemical shifts were referenced to trimethylsilyl propanoic acid (TSP) at δ 0.00 ppm.

Spectral alignment was carried out using the correlation-optimized warping (COW) and interval correlation shifting (icoshift) algorithms. To account for dilution and concentration effects, Probabilistic Quotient Normalization (PQN) was applied to all spectra.

Metabolite identification was performed using Chenomx NMR Suite v8.4 (Chenomx Inc., Edmonton, AB, Canada), the Human Metabolome Database (HMDB, www.hmdb.ca), and reference literature. A single representative signal was selected for each metabolite, as listed in the Supplementary Materials (Table S1—intracellular metabolites; Table S2—extracellular metabolites). This approach follows the generally accepted Metabolomics Standards Initiative (MSI) reporting guidelines and the methodology described by Kostidis et al. [69].

Statistical analysis included 24 extracellular metabolites across all cell lines and intracellular metabolites specific to each cell line: 35 for V79, 37 for SW620, and 36 for MDA-MB-231. Data normality was assessed using the Shapiro–Wilk test. Depending on the data distribution, parametric comparisons were performed using the Student’s *t*-test, while non-parametric data were analyzed using the Mann–Whitney U test. All analyses were conducted in MATLAB, and differences with *p* ≤ 0.05 were considered statistically significant.

#### 4.3.5. Multivariate Data Analysis

Multivariate data analysis was performed using 24 extracellular metabolites obtained from the V79, SW620, and MDA-MB-231 cell lines, as well as intracellular metabolites (35 for V79, 37 for SW620, and 36 for MDA-MB-231). Both multivariate and univariate statistical approaches were applied. A data matrix constructed from metabolite concentrations for each sample was imported into SIMCA-P v17.0 software (Umetrics, Umeå, Sweden) for chemometric evaluation. Prior to analysis, all data were mean-centered and scaled using unit variance (UV) scaling to minimize the influence of concentration magnitude.

Principal Component Analysis (PCA) and Orthogonal Partial Least Squares Discriminant Analysis (OPLS-DA) models were developed to identify clustering patterns and discriminative metabolic features between the experimental groups. Model validation and statistical significance were evaluated using a significance threshold of *p* ≤ 0.05.

## 5. Conclusions

In summary, this in vitro study demonstrated that the combined treatment with metformin and luteolin exerts additive effects on inhibiting the growth of breast and colorectal cancer cells. The combination of both compounds induced alterations in intra- and extracellular metabolite profiles, disrupted cellular energy homeostasis, and suggested V79 Mix suggested an enhancement of glycolytic flux (e.g., decreased extracellular pyruvate). When co-administered with luteolin, metformin increased ROS levels in colorectal cancer cells compared with non-stimulated controls.

These findings suggest that the concurrent use of metformin and luteolin may enhance anticancer efficacy through complementary metabolic and redox mechanisms. Further experimental studies are warranted to confirm the potential of this combination to improve chemosensitivity and support its role as an adjuvant strategy in cancer therapy, especially considering the favorable safety profile, high tolerability, and broad clinical availability of metformin.

Note: “ATP/ADP” denotes a resonance signal corresponding to either ATP or ADP due to spectral overlap observed in the NMR spectrum.

## Figures and Tables

**Table 1:** Representative significantly altered metabolite sets identified by Metabolite Set Enrichment Analysis (MSEA) for SW620 cells incubated with a combination of metformin and luteolin, along with corresponding statistical parameters.

Pathway Name	Hits	Raw *p*	Holm *p*	FDR
Arginine and Proline Metabolism	5	3.12 × 10^−7^	3.06 × 10^−5^	3.06 × 10^−5^
Urea Cycle	4	2.40 × 10^−6^	2.33 × 10^−4^	1.85 × 10^−3^
Mitochondrial Electron Transport Chain	3	5.66 × 10^−5^	5.43 × 10^−3^	1.85 × 10^−3^
Purine Metabolism	4	1.23 × 10^−4^	1.17 × 10^−2^	3.02 × 10^−3^
Ammonia Recycling	3	2.58 × 10^−4^	2.42 × 10^−2^	4.64 × 10^−3^
Citric Acid Cycle	3	2.84 × 10^−4^	2.64 × 10^−2^	4.64 × 10^−3^
Aspartate Metabolism	3	3.73 × 10^−4^	3.43 × 10^−2^	5.22 × 10^−3^

**Table 2:** Representative significantly altered metabolite sets identified by Pathway Analysis (PA) for SW620 cells incubated with a combination of metformin and luteolin, along with corresponding statistical parameters.

Pathway Name	Hits	Raw *p*	−log(*p*)	Holm Adjust	FDR	Impact
Arginine biosynthesis	2	7.23 × 10^−4^	3.14 × 10^0^	5.78 × 10^−2^	5.78 × 10^−2^	0.01
Alanine, aspartate and glutamate metabolism	2	2.95 × 10^−3^	2.53 × 10^0^	2.33 × 10^−1^	1.18 × 10^−1^	0.23
Purine metabolism	2	1.79 × 10^−2^	1.75 × 10^0^	1.00 × 10^0^	4.76 × 10^−1^	0.03
Nicotinate and nicotinamide metabolism	1	4.68 × 10^−2^	1.33 × 10^0^	1.00 × 10^0^	6.31 × 10^−1^	0.03
Histidine metabolism	1	4.98 × 10^−2^	1.33 × 10^0^	1.00 × 10^0^	6.31 × 10^−1^	0.03
Pantothenate and CoA biosynthesis	1	6.20 × 10^−2^	1.21 × 10^0^	1.00 × 10^0^	6.31 × 10^−1^	0.03
Citrate cycle (TCA cycle)	1	6.20 × 10^−2^	1.21 × 10^0^	1.00 × 10^0^	6.31 × 10^−1^	0.03
Beta-Alanine metabolism	1	7.10 × 10^−2^	1.15 × 10^0^	1.00 × 10^0^	6.31 × 10^−1^	0.03
Pyruvate metabolism	1	7.10 × 10^−2^	1.15 × 10^0^	1.00 × 10^0^	6.31 × 10^−1^	0.03
Glycine, serine and threonine metabolism	1	1.09 × 10^−1^	9.82 × 10^−1^	1.00 × 10^0^	7.95 × 10^−1^	0.02
Arginine and proline metabolism	1	1.09 × 10^−1^	9.82 × 10^−1^	1.00 × 10^0^	7.95 × 10^−1^	0.02
Tyrosine metabolism	1	1.27 × 10^−1^	9.88 × 10^−1^	1.00 × 10^0^	8.44 × 10^−1^	0.02

## Data Availability

The original contributions presented in this study are included in the article/Supplementary Material. Further inquiries can be directed to the corresponding authors.

## Supplementary Materials

The following supporting information can be downloaded at: https://www.mdpi.com/article/10.3390/ph18111660/s1, Figure S1: High-resolution ^1^H NMR spectrum (600 MHz) of representative intracellular samples for V-79 cell lines; Figure S2: High-resolution ^1^H NMR spectrum (600 MHz) of representative intracellular samples for SW-620 cell lines; Figure S3: High-resolution ^1^H NMR spectrum (600 MHz) of representative intracellular samples for MDA-MB-231 cell lines; Figure S4: High-resolution ^1^H NMR spectrum (600 MHz) of representative extracellular samples for V-79 cell lines; Figure S5: High-resolution ^1^H NMR spectrum (600 MHz) of representative extracellular samples for SW-620 cell lines; Figure S6: High-resolution ^1^H NMR spectrum (600 MHz) of representative extracellular samples for MDA-MB-231 cell lines; Figure S7: PCA (A) and OPLS-DA (B) score plot of NMR data of all comparison groups of control with different therapeutics in V-79 cell lines; Figure S8: PCA (A) and OPLS-DA (B) score plot of NMR data of all comparison groups of control with different therapeutics in SW-620 cell lines; Figure S9: PCA (A) and OPLS-DA (B) score plot of NMR data of all comparison groups of control with different therapeutics in MDA-MB-231 cell lines; Figure S10: PCA (A) and OPLS-DA (B) score plot of NMR data of all comparison groups of control with different therapeutics in V-79 cell lines (extracellular metabolites); Figure S11: PCA (A) and OPLS-DA (B) score plot of NMR data of all comparison groups of control with different therapeutics in SW-620 cell lines (extracellular metabolites); Figure S12: PCA (A) and OPLS-DA (B) score plot of NMR data of all comparison groups of control with different therapeutics in MDA-MB-231 cell lines (extracellular metabolites); Table S1: Characterization of representative ^1^H NMR signals for intracellular metabolites for V-79, SW-620, and MDA-MB-231 cell lines, were taken for quantification of relative integral used for data analysis; Table S2: Characterization of representative ^1^H NMR signals for extracellular metabolites for V-79, SW-620, and MDA-MB-231 cell lines, were taken for quantification of relative integral used for data analysis; Table S3: Information about graphical representations (intracellular data) for each model for V-79 cell lines. Statistically significant model are marked in orange; Table S4: Information about graphical representations (intracellular data) for each model for SW-620 cell lines; Table S5: Information about graphical representations (intracellular data) for each model for MDA-MB-231 cell lines; Table S6: Information about graphical representations (extracellular data) for each model for V-79 cell lines; Table S7: Information about graphical representations (extracellular data) for each model for SW-620 cell lines; Table S8: Information about graphical representations (extracellular data) for each model for MDA-MB-231 cell lines; Table S9: Relative integration values. expressed as means with standard deviations, and *p*-values from statistical analyses, calculated for metabolites detected by 1H-NMR in cell pellet samples for V-98. SW-620. and MDA-MB-231 cell lines Statistically significant metabolites are marked in green; Table S10: Relative integration values, expressed as means with standard deviations, and *p*-values from statistical analyses, calculated for metabolites detected by ^1^H-NMR in medium samples for V-98, SW-620, and MDA-MB-231 cell lines. Statistically significant metabolites are marked in green.
