# Supplementary material for: Additive Anticancer and Antioxidant Effects of Metformin and Luteolin in Breast and Colorectal Cancer Cell Lines
Source: Pharmaceuticals (Basel). 2025 Nov 1;18(11):1660. doi: 10.3390/ph18111660 (PMC12655200; doi:10.3390/ph18111660)
Supplement: Supplementary file 1 [file pharmaceuticals-18-01660-s001.zip › pharmaceuticals-3921447-supplementary.pdf]

Supplementary data

Table S1. Characterization of representative ^{1}H NMR signals for intracellular metabolites for V-79, SW-620, and MDA-MB-231 cell lines, were taken for quantification of relative integral used for data analysis.

Metabolite	Chemical shift (ppm)	Multiplicity	HMDB ID
2- hydroxyvalerate	0.9	t	HMDB0001863
3-metyl-2- oxovalerate *	1.088	d	HMDB0037114
isoleucine	0.997	d	HMDB0000172
leucine	0.95	t	HMDB0000687
valine	1.03	d	HMDB0000883
lactate	1.32	d	HMDB0000190
alanine	1.47	d	HMDB0001310
lysine	1.7	m	HMDB0000182
arginine	1.64	m	HMDB0000517
acetate	1.91	s	HMDB0000042
glutamate	2.34	m	HMDB0060475
glutamine	2.45	m	HMDB0003423
pyruvate	2.36	s	HMDB0000243
succinate	2.39	s	HMDB0000254
glutathione	2.56	m	HMDB0000125
aspartate	2.67	dd	HMDB0000191
taurine	3.409	t	HMDB0000251
Metahanol **	3.35	s	HMDB0001875
glucose	3.46	m	HMDB0003405
glycine	3.55	s	HMDB0000123
threonine	4,25	m	HMDB0000167
creatine phosphate	3.034	s	HMDB0001511
UDP-glucose	5.22	d	HMDB0000286
ATP/ADP	6.13	s	-
fumarate	6.51	s	HMDB0000134
tyrosine	6.89	d	HMDB0000866
histamine	7.74	s	HMDB0000870

phenylalanine	7.4	m	HMDB0000159
xanthine	7.83	s	HMDB0000292
NAD+/NADP$^+$	8.41	s	-
NAD+	8.16	s	HMDB0000902
AXP (AMP/ATP/ADP)	8.26	s	-
AMP	8.57	s	HMDB0000045
formate	8.44	s	HMDB0000142
ADP	8.52	s	HMDB0001178
unknown	2.7	s	-
myo-inositol	4,06	dd	HMDB0000211
UDP-N-acetyloglucosamine	2.068	s	HMDB0304526
O-phosphocholine	3.21	s	HMDB0001565
carnitine	3.22	s	HMDB0000062
creatine	3,024	s	-

d – doublet, dd – doublet of doublets, s – singlet, t – triplet, m – multiplet; * metabolite not subjected to quantitative analysis due to the lack of a quantifiable unique signal; **the signal from extraction solvent, not subjected to quantitative analysis

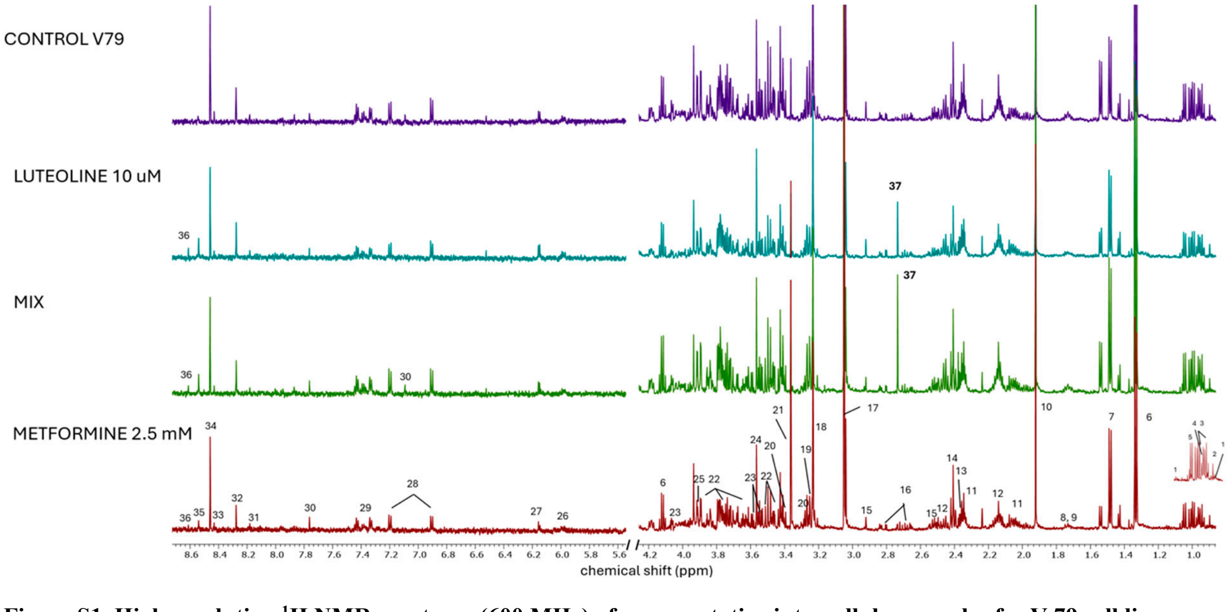

Figure S1. High-resolution ^{1}H NMR spectrum (600 MHz) of representative <u>intracellular samples</u> for V-79 cell lines
1- 3-methyl-2-oxovalerate; 2 - hydroxyvalerate; 3 - isoleucine; 4 – leucine; 5 – valine; 6 – lactate; 7 – alanine; 8 – lysine; 9 -arginine; 10 -acetate; 11 –glutamate; 12 –glutamine; 13 – pyruvate; 14 – succinate; 15 – glutathione; 16 – aspartate; 17 –creatine phosphate; 18 – O-phosphocholine; 19 – carnitine; 20 – taurine; 21 – methanol; 22- glucose; 23-myo-inositol; 24- glycine; 25- creatine; 26- UDP-Glucose; 27 – ATP/ADP; 28 – tyrosine; 29 – phenylalanine; 30 – histamine; 31 – NAD$^+$; 32 – AXP (AMP/ADP/ATP); 33- NAD $^+$/ NADP$^+$; 34 – formate; 35 – ADP; 36 – AMP; 37 – unknown (2.7 ppm)

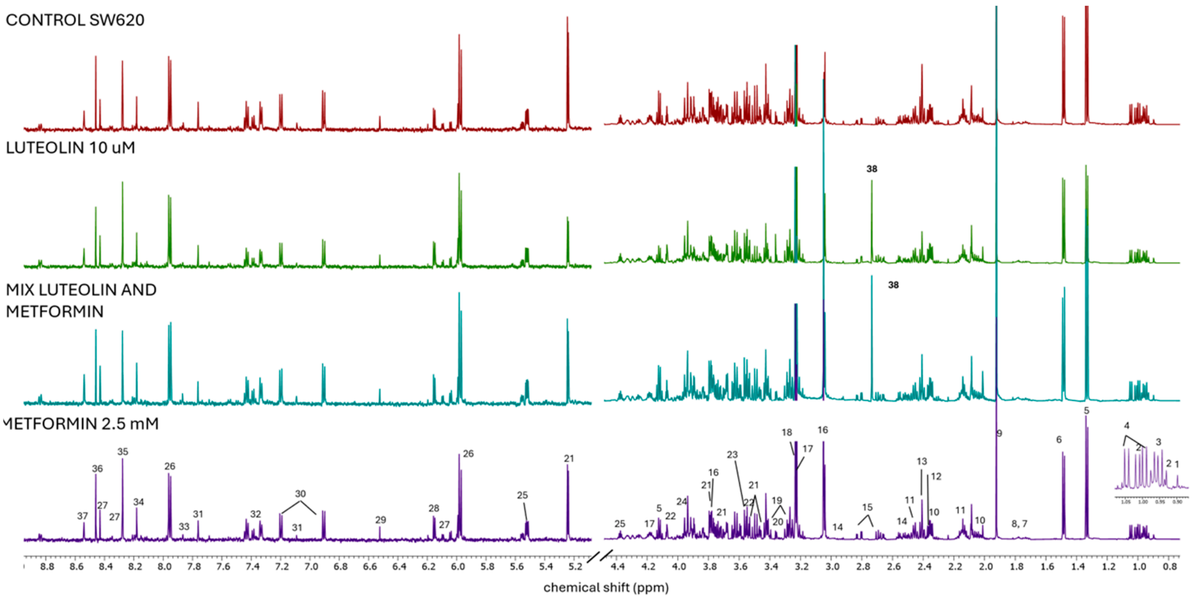

Figure S2. High-resolution ^{1}H NMR spectrum (600 MHz) of representative <u>intracellular samples</u> for SW-620 cell lines
1 - hydroxyvalerate; 2 - isoleucine; 3– leucine; 4 – valine; 5 – lactate; 6 – alanine; 7 – lysine; 8 -arginine; 9 -acetate; 10 –glutamate; 11 –glutamine; 12 – pyruvate; 13– succinate; 14 – glutathione; 15 – aspartate; 16 –creatine phosphate; 17 – O-phosphocholine; 18 – carnitine; 19 – taurine; 20 – methanol; 21- glucose; 22-myo-inositol; 23- glycine; 24- creatine; 25- UDP-N-acetyl glucosamine; 26 – UDP- glucose; 27 –NAD^{+}/$NADP^{+}$; 28 –ATP/ADP; 29 – fumarate; 30-tyrosine; 31- histamine; 32-phenylalanine; 33- xanthine; 34- NAD^{+}; 35-AXP (AMP/ADP/ATP); 36 – formate; 37 – ADP; 38 – unknown (2.7 ppm)

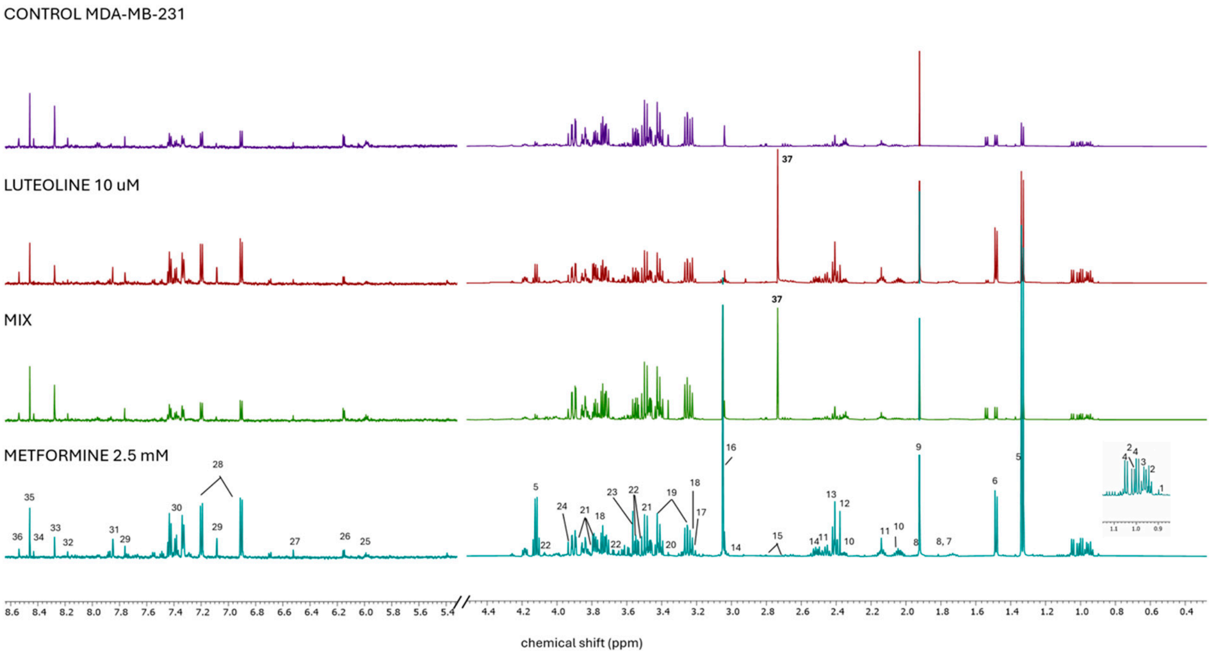

Figure S3. High-resolution ^{1}H NMR spectrum (600 MHz) of representative <u>intracellular samples</u> for MDA-MB-231 cell lines
1 - hydroxyvalerate; 2 - isoleucine; 3– leucine; 4 – valine; 5 – lactate; 6 – alanine; 7 – lysine; 8 -arginine; 9 -acetate; 10 –glutamate; 11 –glutamine; 12 – pyruvate; 13– succinate; 14 – glutathione; 15 – aspartate; 16 –creatine phosphate; 17 – O-phosphocholine; 18 – carnitine; 19 – taurine; 20 – methanol; 21- glucose; 22-myo-inositol; 23- glycine; 24-creatine; 25 – UDP- glucose; 26 – ATP/ADP; 27 – fumarate; 28-tyrosine; 29- histamine; 30-phenylalanine; 31- xanthine; 32- NAD^{+}; 33- AXP (AMP/ADP/ATP); 34 – NAD+/NADP+; 35 – formate; 36 – ADP; 37 – unknown (2.7 ppm)

Table S2. Characterization of representative ^{1}H NMR signals for extracellular metabolites for V-79, SW-620, and MDA-MB-231 cell lines, were taken for quantification of relative integral used for data analysis.

Metabolite	Chemical shift (ppm)	Multiplicity	HMDB ID
isoleucine	0.996	d<br>d	HMDB0000172
leucine	0.95	t	HMDB0000687
valine	1.03	d	HMDB0000883
lactate	1.32	d	HMDB0000190
alanine	1.47	d	HMDB0001310
lysine	1.7	m	HMDB0000182
acetate	1.91	s	HMDB0000042
Glutamate/ pyroglutamate	2.02	m	-
glutamine	2.45	m	HMDB0003423
pyruvate	2.36	s	HMDB0000243
succinate	2.39	s	HMDB0000254
Creatine/ phosphocreatine	3.036	-	-
Metahanol **	3.35	s	HMDB0001875
glucose	3.46	m	HMDB0003405
glycine	3.55	s	HMDB0000123
threonine	4.24	m	HMDB0000167
pyroglutamine	4.17	m	HMDB0000267
tyrosine	6.89	d	HMDB0000866
histamine	7.74	s	HMDB0000870
phenylalanine	7.4	m	HMDB0000159
formate	8.44	s	HMDB0000142
unknown	2.7	s	HMDB0000087
choline	3.19	s	HMDB0000097
imidazole	8.04	s	HMDB0001525
π-methylhistidine	7.14	s	HMDB0000479

d – doublet, dd – doublet of doublets, s – singlet, t – triplet, m – multiplet; **the signal from extraction solvent, not subjected to quantitative analysis

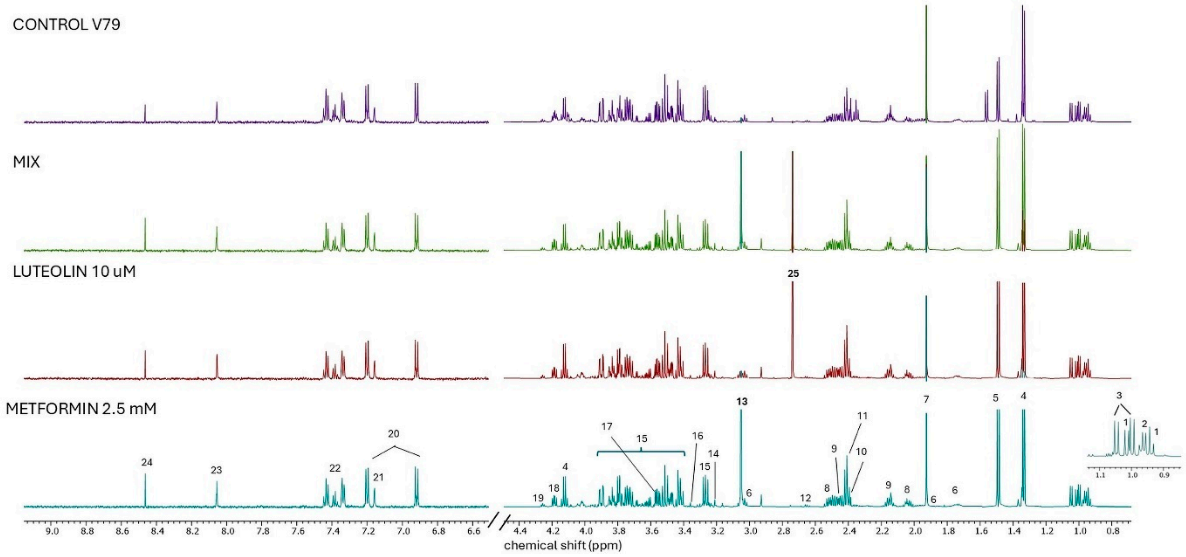

Figure S4. High-resolution ^{1}H NMR spectrum (600 MHz) of representative <u>extracellular samples</u> for V-79 cell lines
1 - isoleucine; 2– leucine; 3- leucine; 4 –lactate; 5 – alanine; 6 – lysine; 7 -acetate; 8–glutamate/ pyroglutamine; 9 –glutamine; 10 – pyruvate; 11– succinate; 12 – methionine; 13 – creatine/creatine phosphate; 14 –choline; 15 – glucose; 16 – methanol; 17- glycine; 18- pyroglutamine; 19 – threonine; 20-tyrosine; 21- π-methylhistidine; 22-phenylalanine; 23- imidazole; 24-formate; 25 –- unknown

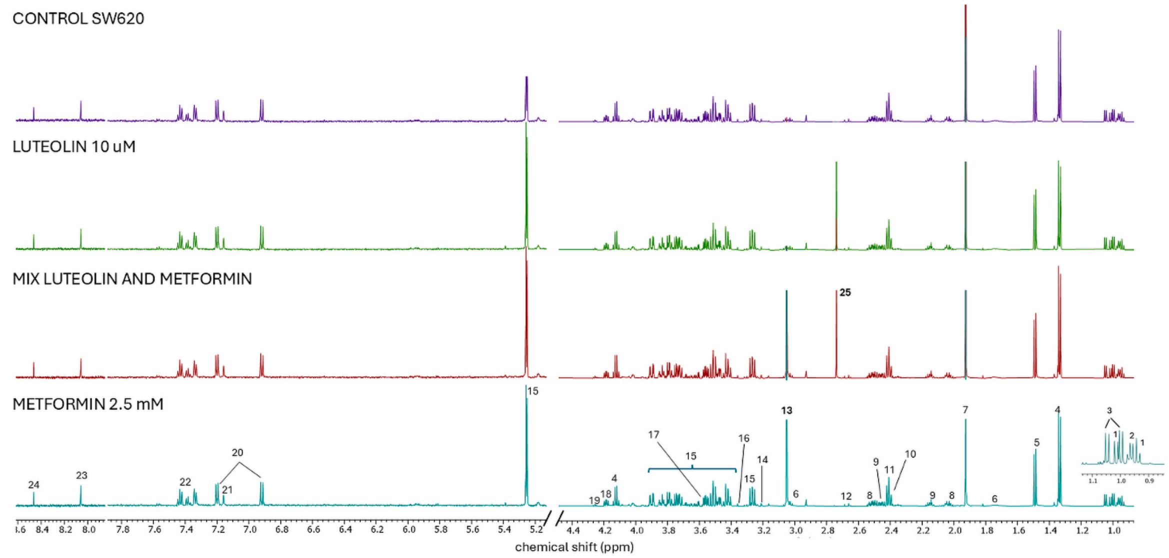

Figure S5. High-resolution 1H NMR spectrum (600 MHz) of representative extracellular samples for SW-620 cell lines
1 - isoleucine; 2 – leucine; 3– valine; 4 –lactate; 5 – alanine; 6 – lysine; 7 -acetate; 8–glutamate/ pyroglutamine; 9 –glutamine; 10 – pyruvate; 11– succinate; 12 – methionine; 13 – creatine/creatine phosphate; 14 –choline; 15 – glucose; 16 – methanol; 17- glycine; 18- pyroglutamine; 19 – threonine; 20-tyrosine; 21- π-methylhistidine; 22-phenylalanine; 23- imidazole; 24-formate; 25 –- unknown

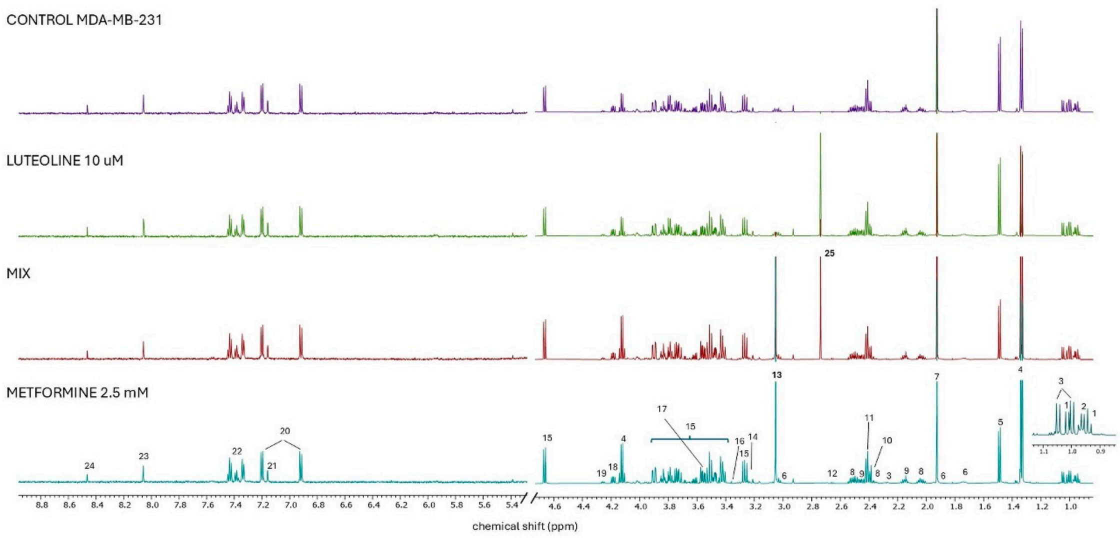

Figure S6. High-resolution ^{1}H NMR spectrum (600 MHz) of representative extracellular samples for MDA-MB-231 cell lines

1 - isoleucine; 2 – leucine; 4– valine; 4 –lactate; 5 – alanine; 6 – lysine; 7 -acetate; 8–glutamate/ pyroglutamine; 9 –glutamine; 10 – pyruvate; 11– succinate; 12 – methionine; 13 – creatine/creatine phosphate; 14- choline; 15 -glucose; 16 – methanol; 17- glycine; 18- pyroglutamine; 19 – threonine; 20-tyrosine; 21- π-methylhistidine; 22-phenylalanine; 23- imidazole; 24-formate; 25 - unknown

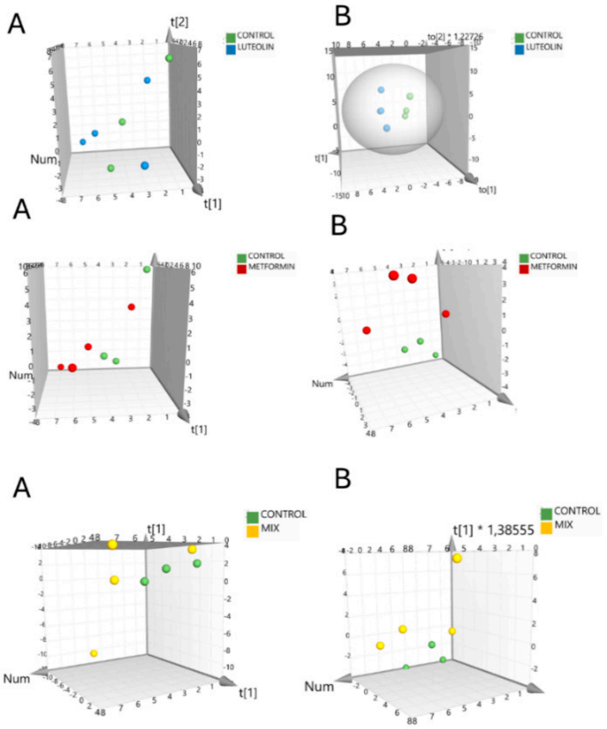

Figure S7. PCA (A) and OPLS-DA (B) score plot of NMR data of all comparison groups of control with different therapeutics in V-79 cell lines

Table S3. Information about graphical representations ***(intracellular data)*** for each model for V-79 cell lines. Statistically significant model are marked in orange.

	**Model**	**Type**	**A**	**N**	**R^2X cum**	**R^2Y cum**	**Q^2 cum**	**CV-ANOVA p-value**
a)	**Control vs luteolin**	PCA-X	2	7	0.641	-	-	-
		OPLS-DA	1+5+0	7	0.98	0.999	1	2.5798e-07
b)	**Control vs metformin**	PCA-X	2	7	0.608	-	-	-
		OPLS-DA	1+0+0	7	0.21	0.75	-0.315	0.1377
c)	**Control vs mix**	PCA-X	1	7	0.481	-	-	-
		OPLS-DA	1+0+0	7	0.326	0.508	-0.686	1

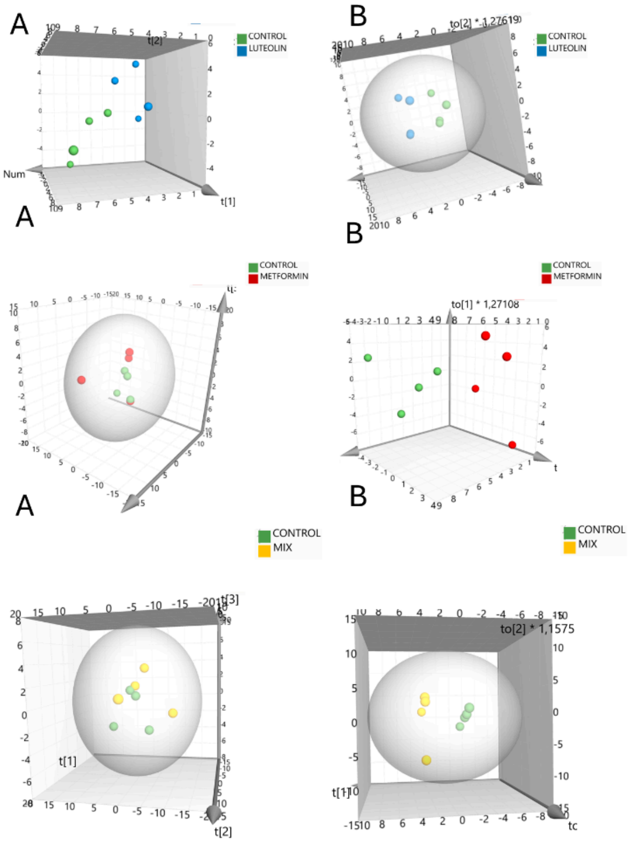

Figure S8. PCA (A) and OPLS-DA (B) score plot of NMR data of all comparison groups of control with different therapeutics in SW-620 cell lines

Table S4. Information about graphical representations ***(intracellular data)*** for each model for SW-620 cell lines.

	Model	Type	A	N	R^2X cum	R^2Y cum	Q^2 cum	CV-ANOVA p-value
a)	**Control vs luteolin**	PCA-X	2	8	0.836	-	-	-
		OPLS-DA	1+4+0	8	0.98	0.994	0.922	0.497968
b)	**Control vs metformin**	PCA-X	3	8	0.903	-	-	-
		OPLS-DA	1+6+0	8	1	0.87	-0.995	0.1377
c)	**Control vs mix**	PCA-X	3	8	0.992	-	-	-
		OPLS-DA	1+1+0	8	0.483	0.716	0.0225	0.991682

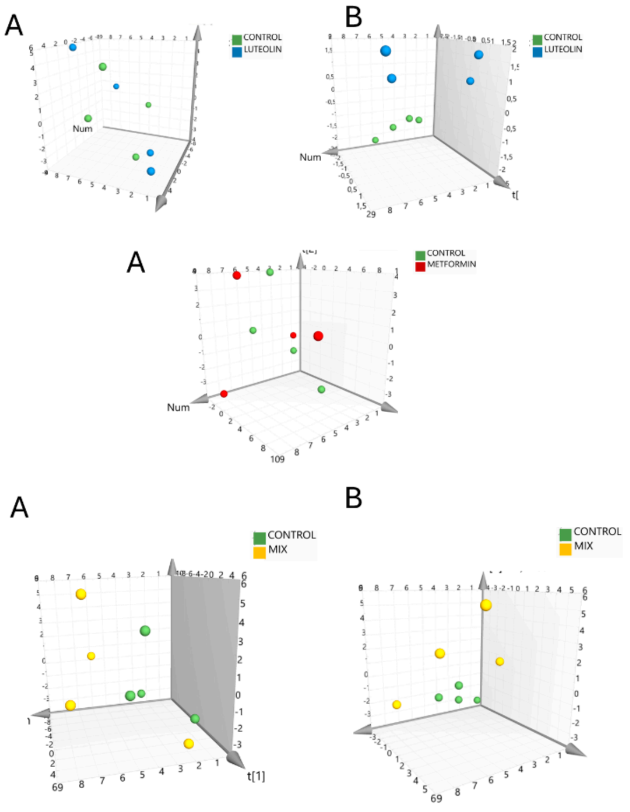

Figure S9. PCA (A) and OPLS-DA (B) score plot of NMR data of all comparison groups of control with different therapeutics in MDA-MB-231 cell lines.

Table S5. Information about graphical representations **(intracellular data)** for each model for MDA-MB-231 cell lines.

	Model	Type	A	N	R^2X cum	R^2Y cum	Q^2 cum	CV-ANOVA p-value
a)	**Control vs luteolin**	PCA-X	2	8	0.74	-	-	-
		OPLS-DA	1+0+0	8	0.0578	0.895	-1.6	1
b)	**Control vs metformin**	PCA-X	2	8	0.736	-	-	-
		OPLS-DA	-	-	-	-	-	-
c)	**Control vs mix**	PCA-X	2	8	0.739	-	-	-
		OPLS-DA	1+0+0	8	0.199	0.508	-0.903	1

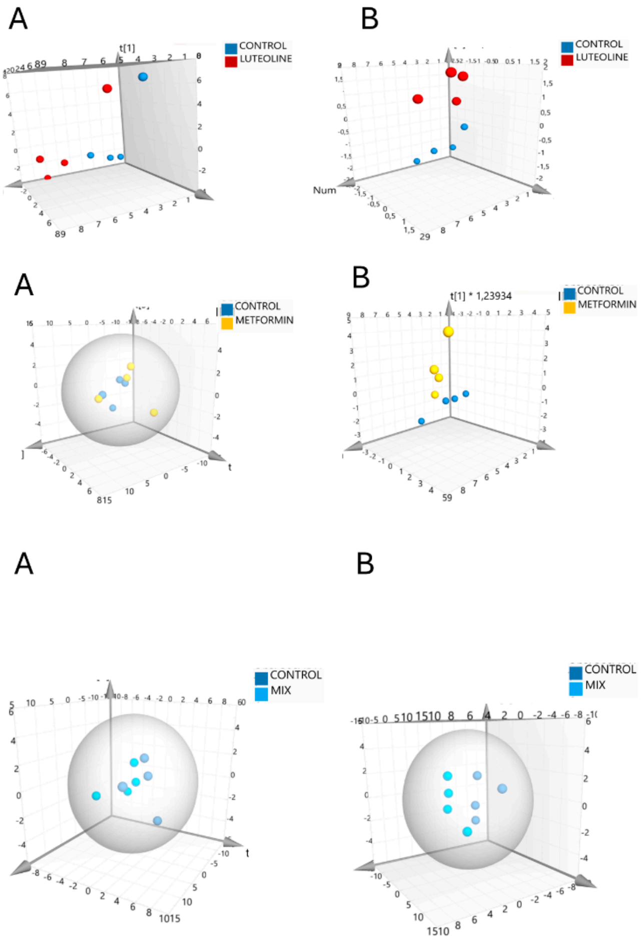

Figure S10. PCA (A) and OPLS-DA (B) score plot of NMR data of all comparison groups of control with different therapeutics in V-79 cell lines (extracellular metabolites).

Table S6. Information about graphical representations **(extracellular data)** for each model for V-79 cell lines.

	Model	Type	A	N	R^2X cum	R^2Y cum	Q^2 cum	CV-ANOVA p-value
a)	**Control vs luteolin**	PCA-X	1	8	0.648	-	-	-
		OPLS-DA	1+0+0	8	0.108	0.883	-0.666	1
b)	**Control vs metformin**	PCA-X	3	7	0.886	-	-	-
		OPLS-DA	1+0+0	8	0.215	0.649	-0.381	0.995
c)	**Control vs mix**	PCA-X	3	8	0.88	-	-	-
		OPLS-DA	1+4+0	8	0.963	0.999	0.933	0.465071

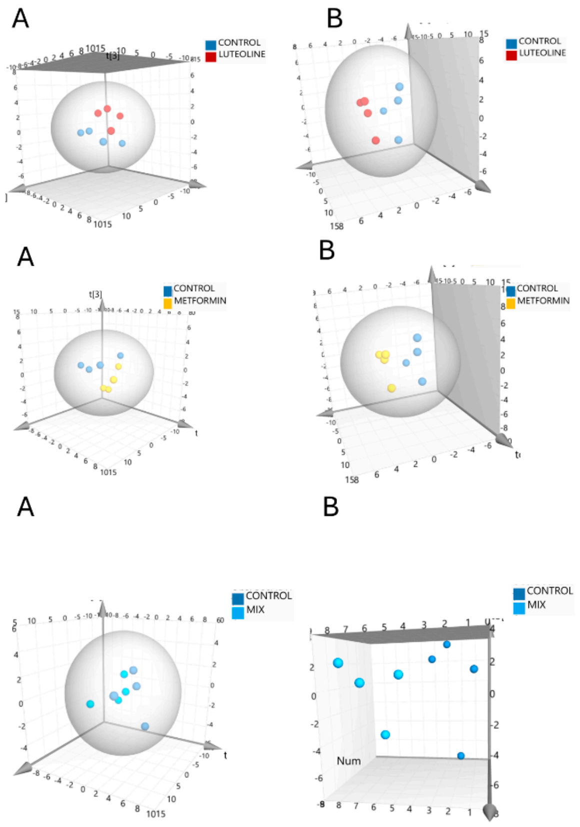

Figure S11. PCA (A) and OPLS-DA (B) score plot of NMR data of all comparison groups of control with different therapeutics in SW-620 cell lines (extracellular metabolites).

Table S7. Information about graphical representations ***(extracellular data)*** for each model for SW-620 cell lines.

	Model	Type	A	N	R^2X cum	R^2Y cum	Q^2 cum	CV-ANOVA p-value
a)	**Control vs luteolin**	PCA-X	3	8	0.868	-	-	-
		OPLS-DA	1+3+0	8	0.898	0.977	0.697	0.534984
b)	**Control vs metformin**	PCA-X	3	8	0.897	-	-	-
		OPLS-DA	1+4+0	8	0.97	1	0.942	0.434593
c)	**Control vs mix**	PCA-X	3	8	0.88	-	-	-
		OPLS-DA	1+1+0	8	0.627	0.949	0.631	0.220785

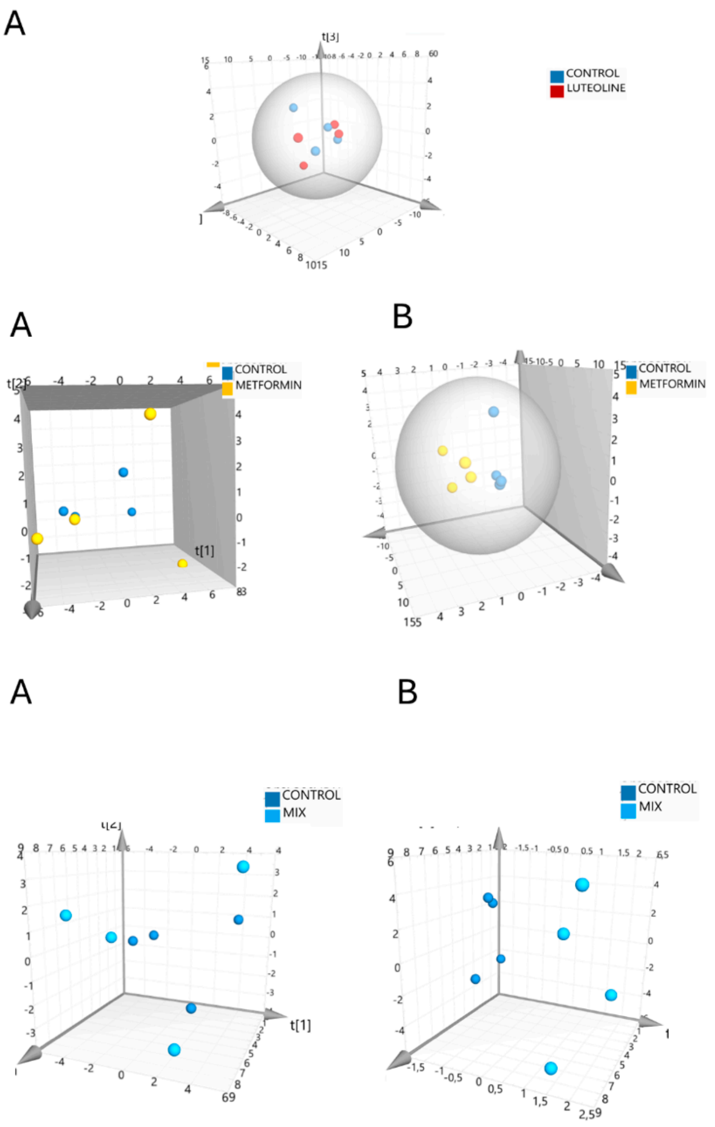

Figure S12. PCA (A) and OPLS-DA (B) score plot of NMR data of all comparison groups of control with different therapeutics in MDA-MB-231 cell lines (extracellular metabolites).

Table S8. Information about graphical representations ***(extracellular data)*** for each model for MDA-MB-231 cell lines.

	Model	Type	A	N	R^2X cum	R^2Y cum	Q^2 cum	CV-ANOVA p-value
a)	**Control vs luteolin**	PCA-X	3	8	0.901	-	-	-
		OPLS-DA	-	-	-	-	-	-
b)	**Control vs metformin**	PCA-X	2	8	0.788	-	-	-
		OPLS-DA	1+2+0	8	0.765	0.955	0.656	0.400064
c)	**Control vs mix**	PCA-X	3	8	0.746	-	-	-
		OPLS-DA	1+1+0	8	0.591	0.972	0.532	0.339223

Table S9. Relative integration values. expressed as means with standard deviations, and p-values from statistical analyses, calculated for metabolites detected by ^{1}H-NMR in *cell pellet samples* for **V-98. SW-620. and MDA-MB-231 cell lines**. Statistically significant metabolites are marked in green.

V-79 CELL LINE												
Metabolites	Median/Mean											
	Control (CTRL) Vs Luteolin (LUT)				Control (CTRL) vs Metformin(METF)				Control (CTRL) vs Mix (MIX)			
	p	CTRL	LUT	SD (SD CTRL/ SD LUT	p	CTRL	METF	SD (SD CTRL/ SD METF	p	CTRL	MIX	SD (SD CTRL/ SD MIX
2-HYDROXYVALERATE	0.38	0.0122	0.0103	0.003/0.002	0.88	0.0122	0.0124	0.003/0.001	0.49	0.0122	0.012	0.003/0.002
ISOLEUCINE	0.3	0.0100	0.0086	0.00068/0.001	0.61	0.0100	0.0117	0.00068/0.004	0.64	0.0100	0.01	0.00068/0.003
LEUCINE	0.18	0.0180	0.0153	0.00068/0.001	0.60	0.0180	0.0207	0.00068/0.008	0.70	0.0180	0.0194	0.00068/0.006
VALINE	0.40	0.0260	0.0219	0.0008/0.0034	0.63	0.0260	0.0295	0.0008/0.011	0.81	0.0260	0.0271	0.0008/0.006
LACTATE	0.43	0.1907	0.1647	0.0263/0.037	0.70	0.1907	0.2105	0.0263/0.068	0.98	0.1907	0.1916	0.0263/0.049
ALANINE	0.26	0.1035	0.0756	0.030/0.018	0.84	0.1035	0.1107	0.030/0.045	0.69	0.1035	0.0939	0.030/0.021
LYSINE	0.69	0.0273	0.0254	0.006/0.004	0.71	0.0273	0.0304	0.006/0.011	0.85	0.0273	0.0261	0.006/0.007
ARGININE	0.60	0.0052	0.0062	0.001/0.003	0.53	0.0052	0.0059	0.001/0.001	0.45	0.0052	0.0038	0.001/0.002
ACETATE	0.11	0.1319	0.0816	0.038/0.017	0.62	0.1319	0.1413	0.038/0.038	0.32	0.1319	0.1007	0.038/0.024
GLUTAMATE	0.47	0.0706	0.0613	0.013/0.013	0.77	0.0706	0.0740	0.013/0.012	0.92	0.0706	0.0721	0.013/0.017
GLUTAMINE	0.83	0.0354	0.0386	0.005/0.020	0.28	0.0354	0.0556	0.005/0.024	0.24	0.0354	0.0475	0.005/0.013
PYRUVATE	0.75	0.0063	0.0069	0.002/0.001	0.51	0.0063	0.0083	0.002/0.004	0.37	0.0063	0.0095	0.002/0.004

**SUCCINATE**	0.18	0.0235	0.0134	0.010/0.004	1.00	0.0235	0.0299	0.010/0.016	0.75	0.0235	0.0260	0.010/0.007
**GLUTATHIONE**	0.99	0.0078	0.0078	0.001/0.001	0.87	0.0078	0.0075	0.001/0.002	0.76	0.0078	0.0082	0.001/0.002
**ASPARTATE**	0.27	0.0064	0.0052	0.001/0.001	0.19	0.0064	0.0047	0.001/0.001	0.88	0.0064	0.0065	0.001/0.001
**PHOSPHOCREATINE**	0.25	0.0069	0.0060	0.001/0.001	0.043	0.0069	0.2768	0.001/0.133	0.022	0.0069	0.2629	0.001/0.215
**O-PHOSPHOCHOLINE**	0.82	0.0276	0.0225	0.001/0.001	0.85	0.0276	0.0208	0.001/0.133	0.86	0.0276	0.0230	0.001/0.101
**CARNITINE**	0.29	0.0776	0.0625	0.0086/0.0172	0.15	0.0776	0.0590	0.0086/0.0140	0.28	0.0776	0.0658	0.0086/0.0122
**TAURINE**	0.3783	0.0136	0.0115	0.002/0.002	0.44	0.0136	0.0200	0.002/0.011	0.85	0.0136	0.0211	0.002/0.018
**GLUCOSE**	0.19	0.0689	0.0526	0.015/0.009	0.34	0.0689	0.1488	0.015/0.122	0.98	0.0326	0.0324	0.015/0.179
**MYO-INOSITOL**	0.8748	0.0326	0.0313	0.012/0.007	0.79	0.0326	0.0349	0.012/0.007	0.98	0.0326	0.0324	0.012/0.007
**GLYCINE**	0.95	0.0282	0.0279	0.006/0.005	0.69	0.0282	0.0300	0.006/0.004	0.3411	0.0282	0.0361	0.006/0.010
**PHOSPHOCREATINE**	0.76	0.0074	0.0071	0.001/0.001	0.22	0.0074	0.0095	0.001/0.002	0.46	0.0074	0.0098	0.001/0.005
**ATP/ADP**	0.91	0.0081	0.0085	0.004/0.002	0.99	0.0081	0.0081	0.004/0.005	0.88	0.0081	0.0086	0.004/0.003
**UDP-GLUCOSE**	0.44	0.0424	0.0519	0.001/0.002	0.91	0.0424	0.0407	0.001/0.006	0.83	0.0424	0.0470	0.001/0.004
**TYROSINE**	0.23	0.0097	0.0076	0.001/0.002	0.87	0.0097	0.0103	0.001/0.005	0.8365	0.0097	0.0104	0.001/0.005
**PHENYLALANINE**	0.24	0.0124	0.0100	0.002/0.002	0.83	0.0124	0.0133	0.002/0.006	0.99	0.0124	0.0124	0.002/0.005
**HISTAMINE**	0.097	0.0027	0.0016	0.0008/0.0003	0.91	0.0027	0.0028	0.0008/0.0013	0.39	0.0027	0.0021	0.0008/0.0005
**FORMATE**	0.012	0.0121	0.0074	0.002/0.004	0.28	0.0121	0.0106	0.002/0.001	0.051	0.0121	0.0094	0.002/0.001
**NAD^+**	0.62	0.0023	0.0020	0.001/0.001	0.72	0.0023	0.0021	0.001/0.001	0.41	0.0023	0.0018	0.001/0.001
**UNKNOWN**	0.04	0.0042	0.0894	0.001/0.002	0.12	0.0042	0.0030	0.001/0.001	0.009	0.0042	0.0412	0.001/0.044
**AXP (ATP/ADP/AMP)**	0.99	0.0088	0.0088	0.005/0.003	0.91	0.0088	0.0084	0.005/0.004	0.82	0.0088	0.0098	0.005/0.004
**NAD+/NADP+**	0.22	0.0018	0.0013	0.0005/0.0004	0.15	0.0018	0.0013	0.0005/0.0002	0.23	0.0018	0.0012	0.0005/0.0005

**ADP**	0.67	0.0048	0.0057	0.0028/0.0017	0.98	0.0048	0.0049	0.0028/0.0026	0.89	0.0048	0.0051	0.0028/0.0014
**AMP**	0.63	0.0016	0.0019	0.0019/0.0007	0.86	0.0016	0.0014	0.0019/0.0010	0.69	0.0016	0.0010	0.0019/0.0003
**SW-620 CELL LINE**												
**2-HYDROXYVALERATE**	0.105	0.0201	0.0168	0.002/0.002	0.58	0.0201	0.0189	0.002/0.003	0.28	0.0201	0.0169	0.002/0.004
**ISOLEUCINE**	0.67	0.0243	0.0220	0.007/0.005	0.59	0.0243	0.0273	0.007/0.006	0.33	0.0243	0.0307	0.007/0.008
**LEUCINE**	0.61	0.0450	0.0407	0.010/0.009	0.74	0.0450	0.0482	0.010/0.012	0.35	0.0450	0.0557	0.010/0.015
**VALINE**	0.61	0.0610	0.0537	0.017/0.015	0.70	0.0610	0.0663	0.017/0.015	0.37	0.0610	0.0756	0.017/0.020
**LACTATE**	0.09	0.4787	0.3485	0.087/0.071	0.53	0.4787	0.4348	0.087/0.071	0.76	0.4787	0.5072	0.087/0.0128
**ALANINE**	0.97	0.2848	0.2821	0.09/0.09	0.69	0.2848	0.3082	0.09/0.04	0.32	0.2848	0.3526	0.09/0.06
**LYSINE**	0.59	0.0407	0.0353	0.013/0.010	0.96	0.0407	0.0411	0.013/0.015	0.423	0.0407	0.0485	0.013/0.009
**ARGININE**	0.36	0.0278	0.0349	0.011/0.006	0.89	0.0278	0.0266	0.011/0.009	0.38	0.0278	0.0374	0.011/0.014
**ACETATE**	0.20	0.6246	0.4222	0.303/0.263	0.62	0.6246	0.4808	0.303/0.382	0.87	0.6246	0.5753	0.303/0.412
**GLUTAMATE**	0.89	0.1947	0.1988	0.055/0.046	0.75	0.1947	0.1780	0.055/0.067	0.88	0.1947	0.2044	0.055/0.093
**GLUTAMINE**	0.93	0.1115	0.1098	0.024/0.020	0.7574	0.1115	0.1033	0.024/0.037	0.66	0.1115	0.1214	0.024/0.028
**PYRUVATE**	0.1401	0.0121	0.0080	0.003/0.003	0.72	0.0121	0.0133	0.003/0.003	0.42	0.0121	0.0144	0.003/0.003
**SUCCINATE**	0.48	0.0749	0.0585	0.024/0.021	0.48	0.0749	0.0617	0.024/0.029	0.69	0.0749	0.0616	0.024/0.037
**GLUTATHIONE**	0.6857	0.0124	0.0148	0.005/0.005	0.84	0.0124	0.0132	0.005/0.004	0.52	0.0124	0.0155	0.005/0.006
**ASPARTATE**	0.09	0.0153	0.0227	0.005/0.005	0.16	0.0153	0.0233	0.005/0.007	0.03	0.0153	0.0219	0.005/0.002
**PHOSPHOCREATINE**	0.20	0.0895	0.1100	0.021/0.013	0.04	0.0895	0.4274	0.021/0.174	0.02	0.0895	0.4974	0.021/0.0987
**O-PHOSPHOCHOLINE**	0.28	0.2786	0.3435	0.061/0.073	0.66	0.2786	0.3065	0.061/0.073	0.61	0.2786	0.3026	0.061/0.048

**CARNITINE**	0.39	0.4943	0.6166	0.171/0.152	0.86	0.4943	0.5179	0.171/0.160	0.36	0.4943	0.6630	0.171/0.242
**TAURINE**	0.80	0.1164	0.1118	0.021/0.036	0.51	0.1164	0.1329	0.021/0.023	0.34	0.1164	0.1528	0.021/0.056
**GLUCOSE**	0.22	0.1853	0.1514	0.019/0.039	0.59	0.1853	0.2150	0.019/0.085	0.54	0.1853	0.2434	0.019/0.144
**MYO-INOSITOL**	0.23	0.1209	0.1592	0.037/0.034	0.84	0.1209	0.1272	0.037/0.035	0.38	0.1209	0.1526	0.037/0.045
**GLICINE**	0.28	0.0478	0.0589	0.014/0.009	0.22	0.0478	0.0701	0.014/0.025	0.14	0.0478	0.0824	0.014/0.033
**PHOSPHOCREATINE**	0.20	0.0489	0.0637	0.012/0.016	0.89	0.0489	0.0477	0.012/0.010	0.67	0.0489	0.0559	0.012/0.015
**UDP-GLUCOSE**	0.40	0.0571	0.0691	0.014/0.018	0.92	0.0571	0.0556	0.014/0.021	0.87	0.0571	0.0606	0.014/0.033
**ATP/ADP**	0.21	0.0125	0.0169	0.002/0.005	0.06	0.0125	0.0180	0.002/0.005	0.03	0.0125	0.0199	0.002/0.003
**TYROSINE**	0.73	0.0224	0.0207	0.006/0.006	0.45	0.0224	0.0263	0.006/0.006	0.25	0.0224	0.0310	0.006/0.010
**PHENYLALANINE**	0.73	0.0274	0.0254	0.007/0.006	0.62	0.0274	0.0304	0.006/0.007	0.33	0.0274	0.0356	0.006/0.012
**HISTAMINE**	0.49	0.0048	0.0057	0.006/0.001	0.84	0.0048	0.0045	0.006/0.002	0.84	0.0048	0.0044	0.006/0.003
**FORMATE**	0.69	0.0110	0.0101	0.003/0.002	0.33	0.0110	0.0084	0.003/0.002	0.47	0.0110	0.0091	0.003/0.002
**NAD+**	0.20	0.0086	0.0112	0.002/0.003	0.86	0.0086	0.0090	0.002/0.003	0.39	0.0086	0.0114	0.003/0.006
**UNKNOWN**	0.03	0.0025	0.1287	0.0006/0.020	0.48	0.0025	0.0031	0.0006/0.0039	0.03	0.0025	0.1221	0.0006/0.0126
**ATP/AMP/ADP (AXP)**	0.31	0.0167	0.0205	0.002/0.005	0.20	0.0167	0.0197	0.002/0.002	0.08	0.0167	0.0206	0.002/0.003
**NAD+/NADP**	0.22	0.0060	0.0074	0.001/0.002	0.85	0.0060	0.0062	0.001/0.002	0.52	0.0060	0.0071	0.001/0.003
**FUMARATE**	0.43	0.0012	0.0015	0.0006/0.0002	0.051	0.0012	0.0022	0.0006/0.0004	0.03	0.0012	0.0029	0.0006/0.0004
**UDP-N-ACETYLGLUKOZAMINE**	0.48	0.0586	0.0695	0.018/0.020	0.84	0.0586	0.0547	0.018/0.026	0.85	0.0586	0.0629	0.018/0.034
**ADP**	0.36	0.0077	0.0088	0.0003/0.0018	0.26	0.0077	0.0099	0.0003/0.0027	0.04	0.0077	0.0120	0.0003/0.0026
**XANTHINE**	0.34	0.0040	0.0036	0.002/0.002	0.88	0.0040	0.0033	0.002/0.003	0.48	0.0040	0.0052	0.002/0.003
**MDA-MB-231 CELL LINE**												

**2-HYDROXYVALERATE**	0.88	0.0139	0.0126	0.002/0.005	0.48	0.0139	0.0151	0.002/0.003	0.68	0.0139	0.0158	0.002/0.004
**ISOLEUCINE**	0.94	0.0205	0.0200	0.008/0.007	0.97	0.0205	0.0208	0.008/0.011	0.95	0.0205	0.0201	0.008/0.008
**LEUCINE**	0.89	0.0350	0.0367	0.014/0.014	0.99	0.0350	0.0352	0.014/0.019	0.98	0.0350	0.0349	0.014/0.015
**VALINE**	0.96	0.0501	0.0494	0.020/0.017	0.90	0.0501	0.0527	0.020/0.030	0.9739	0.0501	0.0508	0.020/0.024
**LACTATE**	0.85	0.3347	0.3112	0.176/0.124	0.50	0.3347	0.5596	0.176/0.521	0.50	0.3347	0.5355	0.176/0.461
**ALANINE**	0.88	0.1821	0.1694	0.115/0.094	0.48	0.1821	0.1702	0.115/0.115	0.8857	0.1821	0.1722	0.115/0.105
**LYSINE**	0.88	0.0509	0.0500	0.014/0.018	0.93	0.0509	0.0494	0.014/0.018	0.90	0.0509	0.0495	0.014/0.013
**ARGININE**	0.20	0.0090	0.0055	0.0018/0.0026	0.29	0.0090	0.0063	0.0018/0.0038	0.31	0.0090	0.0072	0.0018/0.0023
**ACETATE**	0.90	0.2944	0.2750	0.219/0.165	1.00	0.2944	0.3041	0.219/0.245	0.99	0.2944	0.2966	0.219/0.206
**GLUTAMATE**	0.96	0.1001	0.1011	0.035/0.026	0.81	0.1001	0.1091	0.035/0.051	0.20	0.1001	0.1129	0.035/0.037
**GLUTAMINE**	0.89	0.0655	0.0606	0.044/0.044	0.99	0.0655	0.0657	0.044/0.046	0.9	0.0655	0.0652	0.044/0.044
**PYRUVATE**	0.68	0.0119	0.0158	0.009/0.012	1.00	0.0119	0.0259	0.009/0.033	0.68	0.0119	0.0248	0.009/0.028
**SUCCINATE**	0.93	0.0510	0.0482	0.039/0.036	0.87	0.0510	0.0569	0.039/0.046	0.93	0.0510	0.0537	0.039/0.042
**GLUTATHIONE**	0.76	0.0124	0.0130	0.002/0.002	0.29	0.0124	0.0154	0.002/0.004	0.25	0.0124	0.0149	0.002/0.003
**ASPARTATE**	0.93	0.0136	0.0132	0.006/0.004	0.503	0.0136	0.0104	0.006/0.004	0.75	0.0136	0.0122	0.006/
**PHOSPHOCREATINE**	0.65	0.0646	0.0603	0.012/0.010	0.01	0.0646	0.4711	0.012/0.109	0.02	0.0646	0.4815	0.012/0.090
**O-PHOSPHOCHOLINE**	0.645	0.1615	0.1829	0.0414/0.0655	0.20	0.1615	0.1086	0.0414/0.0493	0.50	0.1615	0.1317	0.0414/0.0590
**CARNITINE**	0.93	0.0555	0.0572	0.020/0.025	0.99	0.0555	0.0554	0.020/0.03	0.84	0.0555	0.0602	0.020/0.035
**TAURINE**	0.99	0.0277	0.0277	0.012/0.011	0.81	0.0277	0.0305	0.012/0.016	0.65	0.0277	0.0335	0.012/0.016
**GLUCOSE**	0.92	0.2342	0.2451	0.139/0.152	0.86	0.2342	0.2569	0.139/0.152	0.77	0.2342	0.2776	0.139/0.206
**MYO-INOSITOL**	0.37	0.0483	0.0555	0.011/0.007	0.88	0.0483	0.0503	0.011/0.021	0.53	0.0483	0.0549	0.011/0.013
**GLICINE**	0.97	0.0454	0.0451	0.010/0.007	0.81	0.0454	0.0496	0.010/0.028	0.19	0.0454	0.0619	0.010/0.017

**PHOSPHOCREATINE**	0.81	0.0062	0.0059	0.002/0.001	0.76	0.0062	0.0057	0.002/0.002	0.68	0.0062	0.0063	0.002/0.001
**UDP-GLUCOSE**	0.88	0.0514	0.0519	0.002/0.001	0.80	0.0514	0.0507	0.002/0.001	0.055	0.0514	0.0470	0.002/0.007
**ATP/ADP**	0.63	0.0087	0.0105	0.005/0.004	0.77	0.0087	0.0077	0.005/0.004	0.67	0.0087	0.0108	0.005/0.006
**FUMARATE**	0.56	0.0022	0.0025	0.006/0.0012	0.05	0.0022	0.0023	0.006/0.0012	0.03	0.0022	0.0021	0.006/0.0014
**TYROSINE**	0.88	0.0171	0.0191	0.008/0.008	0.90	0.0171	0.0182	0.008/0.013	0.77	0.0171	0.0191	0.008/0.009
**PHENYLALANINE**	0.60	0.0218	0.0256	0.008/0.009	0.87	0.0218	0.0233	0.008/0.014	0.73	0.0218	0.0245	0.008/0.011
**HISTAMINE**	0.47	0.0031	0.0048	0.002/0.009	0.99	0.0031	0.0031	0.002/0.003	1	0.0031	0.0033	0.002/0.004
**FORMATE**	0.48	0.0101	0.0087	0.002/0.004	0.89	0.0101	0.0101	0.002/0.002	0.88	0.0101	0.0108	0.002/0.003
**NAD+**	0.65	0.0016	0.002	0.001/0.001	0.97	0.0016	0.0016	0.001/0.001	0.48	0.0016	0.0021	0.001/0.001
**UNKNOWN**	0.03	0.0556	0.1611	0.08/0.16	0.32	0.0556	0.004	0.08/0.02	0.04	0.0556	0.1425	0.08/0.11
**ATP/AMP /ADP(AXP)**	0.68	0.01	0.0108	0.004/0.003	1	0.01	0.0092	0.004/0.003	0.88	0.01	0.0112	0.004/0.003
**NAD+/NADP**	0.57	0.0014	0.001	0.001/0.001	0.96	0.0014	0.0015	0.001/0.001	0.66	0.0014	0.0017	0.001/0.001
**ADP**	0.30	0.0054	0.0076	0.002/0.003	0.98	0.0054	0.0053	0.002/0.003	0.55	0.0054	0.007	0.002/0.004
**XANTHINE**	0.35	0.0033	0.0054	0.002/0.003	0.99	0.0033	0.0033	0.002/0.004	0.68	0.0033	0.004	0.002/0.003

Table S10. Relative integration values, expressed as means with standard deviations, and p-values from statistical analyses, calculated for metabolites detected by ^{1}H-NMR <u>in *medium samples*</u> for **V-98, SW-620, and MDA-MB-231 cell lines**. Statistically significant metabolites are marked in green.

V-79 CELL LINE												
Metabolites	Median/Mean											
	Control (CTRL) Vs Luteolin (LUT)				Control (CTRL) vs Metformin(METF)				Control (CTRL) vs Mix (MIX)			
	p	CTRL	LUT	SD (SD CTRL/ SD LUT	p	CTRL	METF	SD (SD CTRL/ SD METF	p	CTRL	MIX	SD (SD CTRL/ SD MIX
ISOLEUCINE	1.00	0.2533	0.2599	0.018/0.019	0.68	0.2533	0.2486	0.018/0.030	0.68	0.2533	0.2437	0.018/0.030
LEUCINE	0.68	0.9042	0.8815	0.086/0.088	0.48	0.9042	0.8686	0.086/0.088	0.20	0.9042	0.8458	0.086/0.131
VALINE	0.92	0.9349	0.9254	0.116/0.105	0.89	0.9349	0.9183	0.116/0.155	0.68	0.9349	0.9036	0.116/0.164
ALANINE	0.93	3.0646	3.1718	1.582/1.391	0.89	3.0646	3.2461	1.582/1.615	0.93	3.0646	2.9568	1.582/1.615
LYSINE	0.33	1.0242	0.9744	0.055/0.061	0.37	1.0242	0.9693	0.055/0.061	0.04	1.0242	0.9369	0.055/0.081
ACETATE	0.88	2.6493	2.5424	0.061/0.869	0.68	2.6493	3.0942	0.061/0.719	1.00	2.6493	2.6832	0.061/0.802
GLUTAMATE/PYRO GLUTAMATE	0.99	0.9000	0.8987	0.313/0.243	0.68	0.9000	0.9237	0.313/0.243	1.00	0.9000	0.8841	0.313/0.243
GLUTAMINE	0.6147	2.2449	2.2027	0.066/0.03	0.52	2.2449	2.1534	0.066/0.016	0.04	2.2449	2.1277	0.066/0.026
PYRUVATE	0.13	0.3003	0.2227	0.062/0.048	0.56	0.3003	0.2719	0.062/0.052	0.16	0.3003	0.2233	0.062/0.052
SUCCINATE	0.48	1.2661	1.2876	0.0398/0.06	1.00	1.2661	1.2736	0.0398/0.04	1.00	1.2661	1.2347	0.0398/0.07
METHIONINE	0.45	0.1665	0.1928	0.023/0.052	0.52	0.1665	0.1771	0.023/0.016	0.33	0.1665	0.2177	0.023/0.012
CREATINE/PHOSPH OCREATINE	1.00	0.4962	0.4926	0.093/0.073	0.03	0.4962	10.0459	0.093/0.073	0.02	0.4962	10.0185	0.093/0.063
CHOLINE	0.76	0.2700	0.2585	0.040/0.049	0.97	0.2700	0.2688	0.040/0.041	0.63	0.2700	0.2551	0.040/0.042

**GLUCOSE**	0.65	1.2211	1.2004	0.151/0.141	0.04	1.2211	1.1483	0.151/0.111	0.01	1.2211	1.1429	0.151/0.132
**GLYCINE**	0.55	0.2261	0.2095	0.019/0.007	0.68	0.2261	0.2297	0.019/0.015	0.53	0.2261	0.2081	0.019/0.015
**THREONINE**	0.68	0.2765	0.2633	0.045/0.030	0.92	0.2765	0.2800	0.045/0.039	0.48	0.2765	0.2557	0.045/0.039
**TYROSINE**	0.82	0.2724	0.2677	0.027/0.022	0.79	0.2724	0.2655	0.027/0.035	0.35	0.2724	0.2536	0.027/0.035
**π-METYLHISTYDINE**	0.94	0.0972	0.0980	0.015/0.013	0.88	0.0972	0.0950	0.015/0.020	0.62	0.0972	0.0917	0.015/0.020
**PHENYLOALANINE**	0.88	0.3534	0.3533	0.016/ 0.015	0.88	0.3534	0.3445	0.016/ 0.15	0.02	0.3534	0.3311	0.016/ 0.021
**IMIDAZOLE**	0.87	0.133	0.143	0.003/0.001	0.62	0.133	0.132	0.003/0.001	0.055	0.133	0.125	0.003/0.001
**FORMATE**	0.87	0.0300	0.0315	0.013/ 0.009	0.20	0.0300	0.0342	0.013/ 0.014	0.88	0.0300	0.0292	0.013/ 0.014
**UNKNOWN**	0.028	0.0245	5.0985	0.004/ 0.146	0.34	0.0245	0.0683	0.004/ 0.045	0.02	0.0245	4.7222	0.004/ 0.045
**LACTATE**	0.501	1.6042	1.7347	0.229/ 0.228	0.43	1.6042	1.7418	0.229/ 0.168	0.72	1.6042	1.5390	0.229/ 0.218
**PYROGLUTAMATE**	0.46	1.4849	1.4142	0.091/ 0.127	0.69	1.4849	1.5338	0.091/ 0.127	0.34	1.4849	1.6491	0.091/ 0.182
**SW-620 CELL LINE**												
**ISOLEUCINE**	0.31	0.3863	0.3667	0.029/ 0.010	0.19	0.3863	0.3591	0.029/ 0.014	0.19	0.3863	0.3580	0.029/ 0.020
**LEUCINE**	0.69	1.3606	1.3265	0.126/ 0.069	0.24	1.3606	1.2526	0.126/ 0.069	0.95	1.3606	1.3552	0.126/ 0.069
**VALINE**	0.63	1.4502	1.3924	0.166/0.116	0.39	1.4502	1.3490	0.166/0.088	0.82	1.4502	1.4261	0.166/0.088
**ALANINE**	0.79	5.7976	5.4490	0.299/0.32	0.66	5.7976	5.2396	0.299/0.36	0.86	5.7976	5.5693	0.299/0.42
**LYSINE**	0.46	1.4659	1.4271	0.059/0.062	0.20	1.4659	1.3741	0.059/0.066	0.27	1.4659	1.4152	0.059/0.071
**ACETATE**	0.82	9.5921	8.5757	0.168/0.126	0.94	9.5921	9.2127	0.168/0.126	0.99	9.5921	9.5761	0.168/0.134
**GLUTAMATE/PYRO GLUTAMATE**	0.79	1.8223	1.7206	0.509/0.376	0.68	1.8223	1.5969	0.509/0.375	0.94	1.8223	1.7951	0.509/0.376
**GLUTAMINE**	0.49	2.8844	2.7899	0.135/0.182	0.23	2.8844	2.6970	0.135/0.204	0.54	2.8844	2.9776	0.135/0.204

**PYRUVATE**	0.02	0.2414	0.1879	0.024/0.018	0.49	0.2414	0.2255	0.024/0.029	0.018	0.2414	0.1735	0.024/0.029
**SUCCINATE**	0.7354	2.3895	2.2471	0.545/0.434	0.52	2.3895	2.1345	0.545/0.434	0.80	2.3895	2.2853	0.545/0.366
**METHIONINE**	0.0524	0.3152	0.4461	0.065/0.068	0.78	0.3152	0.3034	0.065/0.034	0.08	0.3152	0.3995	0.065/0.034
**CREATINE/PHOSPH OCREATINE**	0.3429	0.7365	0.6387	0.142/0.227	0.000 031	0.7365	13.7917	0.142/0.373	0.000 8	0.7365	17.2993	0.142/0.673
**CHOLINE**	0.7185	0.4355	0.4146	0.082/0.050	0.64	0.4355	0.4099	0.082/0.041	0.66	0.4355	0.4109	0.082/0.043
**GLUCOSE**	0.4782	4.9933	4.7338	0.528/0.273	0.25	4.9933	4.5490	0.528/0.284	0.53	4.9933	4.7761	0.528/0.219
**GLYCINE**	0.6194	0.3241	0.3060	0.046/0.038	0.68	0.3241	0.3095	0.046/0.037	1.00	0.3241	0.3214	0.046/0.035
**THREONINE**	0.9881	0.4230	0.4236	0.051/0.054	0.6	0.4230	0.3986	0.051/0.054	0.89	0.4230	0.4163	0.051/0.056
**TYROSINE**	0.9900	0.3906	0.3908	0.024/0.012	0.65	0.3906	0.3801	0.024/0.029	0.41	0.3906	0.4073	0.024/0.022
**π-METYLHISTYDINE**	0.7941	0.1602	0.1573	0.015/0.011	0.35	0.1602	0.1479	0.015/0.015	1.00	0.1602	0.1626	0.015/0.012
**PHENYLOALANINE**	0.4049	0.5082	0.4836	0.019/0.043	0.37	0.5082	0.4875	0.019/0.043	0.85	0.5082	0.5118	0.019/0.027
**FORMATE**	0.8392	0.0307	0.0293	0.008/0.008	0.78	0.0307	0.0290	0.008/0.005	0.978	0.0307	0.0309	0.008/0.006
**UNKNOWN**	0.0004	0.0803	7.4817	0.065/0.010	0.44	0.0803	0.0468	0.065/0.010	0.000 8	0.0803	7.7170	0.065/0.555
**IMIDAZOLE**	0.4857	0.17111	0.17754	0.029/0.033	0.69	0.17111	0.18575	0.029/0.023	0.69	0.17111	0.18733	0.029/0.043
**LACTATE**	0.4857	2.2876	2.2719	0.396/0.124	0.35	2.2876	2.1460	0.396/0.083	0.48	2.2876	2.3015	0.396/0.124
**PYROGLUTAMATE**	0.4857	1.9380	2.0466	0.175/0.477	0.61	1.9380	1.8352	0.175/0.276	0.34	1.9380	2.0026	0.175/0.396
**MDA-MB-231 CELL LINE**												
**ISOLEUCINE**	0.97	0.3464	0.3453	0.028/0.052	0.83	0.3464	0.3392	0.028/0.051	0.26	0.3464	0.3106	0.028/0.041
**LEUCINE**	0.76	1.1598	1.1086	0.158/0.231	0.70	1.1598	1.1027	0.158/0.231	0.62	1.1598	1.0892	0.158/0.211
**VALINE**	0.95	1.2460	1.2584	0.209/0.328	0.95	1.2460	1.2320	0.209/0.288	0.99	1.2460	1.2453	0.209/0.252
**ALANINE**	0.91	4.4716	4.7097	0.222/0.348	0.86	4.4716	4.1712	0.222/0.348	0.76	4.4716	3.9432	0.222/0.148

**LYSINE**	0.79	1.3486	1.3181	0.100/0.165	0.65	1.3486	1.2902	0.100/0.165	0.52	1.3486	1.2671	0.100/0.165
**ACETATE**	0.92	3.9603	4.2267	0.413/0.333	0.86	3.9603	4.3808	0.413/0.183	0.98	3.9603	4.0159	0.413/0.527
**GLUTAMATE/PYRO GLUTAMATE**	0.89	1.1780	1.3176	0.632/0.716	0.69	1.1780	1.2354	0.632/0.526	0.69	1.1780	1.1924	0.632/0.528
**GLUTAMINE**	0.46	3.0137	3.4577	0.157/0.0903	0.59	3.0137	3.4506	0.157/0.159	0.65	3.0137	3.3482	0.157/0.257
**PYRUVATE**	0.48	0.3832	0.3447	0.063/0.065	0.65	0.3832	0.4445	0.063/0.213	0.58	0.3832	0.3369	0.063/0.213
**SUCCINATE**	0.88	1.5203	1.7398	0.605/0.670	1.00	1.5203	1.6406	0.605/0.530	0.89	1.5203	1.6176	0.605/0.470
**METHIONINE**	0.71	0.2328	0.2173	0.032/0.061	0.71	0.2328	0.2224	0.032/0.035	0.13	0.2328	0.3062	0.032/0.066
**CREATINE/PHOSPH OCREATINE**	0.64	0.5906	0.7291	0.333/0.359	0.001 1	0.5906	13.9124	0.333/0.579	0.001 1	0.5906	13.6528	0.333/0.308
**CHOLINE**	0.92	0.3670	0.3588	0.076/0.117	0.92	0.3670	0.3742	0.076/0.090	0.75	0.3670	0.3457	0.076/0.079
**GLUCOSE**	0.72	4.1697	4.0738	0.411/0.203	0.58	4.1697	4.4646	0.411/0.206	0.73	4.1697	4.3519	0.411/0.303
**GLYCINE**	0.85	0.2727	0.2620	0.057/0.082	0.88	0.2727	0.3305	0.057/0.164	0.69	0.2727	0.3384	0.057/0.105
**THREONINE**	0.85	0.3638	0.3738	0.069/0.056	0.85	0.3638	0.3513	0.069/0.088	0.87	0.3638	0.3534	0.069/0.084
**TYROSINE**	0.98	0.3680	0.3673	0.045/0.067	0.85	0.3680	0.3775	0.045/0.073	0.73	0.3680	0.3869	0.045/0.077
**π-METYLHISTYDINE**	0.96	0.1382	0.1374	0.021/0.028	0.69	0.1382	0.1306	0.021/0.023	0.69	0.1382	0.1312	0.021/0.022
**PHENYLOALANINE**	0.90	0.4663	0.4615	0.037/0.054	0.90	0.4663	0.4722	0.037/0.074	0.99	0.4663	0.4668	0.037/0.067
**IMIDAZOLE**	0.88	0.141	0.139	0.054/0.056	0.72	0.141	0.136	0.054/0.074	0.79	0.141	0.137	0.054/0.056
**FORMATE**	0.41	0.0161	0.0205	0.006/0.007	0.69	0.0161	0.0183	0.006/0.007	0.79	0.0161	0.0174	0.006/0.005
**UNKNOWN**	0.03	0.0340	4.8959	0.005/0.235	0.78	0.0340	0.0390	0.005/0.027	0.02	0.0340	4.1337	0.005/0.525
**LACTATE**	0.98	2.4609	2.4549	0.254/0.332	0.58	2.4609	2.8640	0.254/0.698	0.68	2.4609	2.7388	0.254/0.698
**PYROGLUTAMATE**	0.89	1.9610	2.0258	0.838/0.421	1.00	1.9610	1.9001	0.838/0.421	1.00	1.9610	1.8213	0.838/0.519

BLANK (Fresh MEM)

Metabolite	concentration
Isoleucine	0.27
Leucine	0.90
Valine	0.94
Alanine	1.97
Lysine	1.03
Acetate	0,22
Glutamate/Pyroglutamate	0.21
Glutamine	2.86
Pyruvate	0.013
Succinate	0.27
Methionine	0.16
Creatine	0.52
Choline	0.3
Methanol	0.001
Glycine	0.13
Threonine	0.27
Tyrosine	0.28
Π-methylhistidine	0.11
Phenylalanine	0.35
Formate	0.009
Unknown	0.001
Glucose	1.43
Lactate	1.86
Pyroglutamate	2.67

BLANK (Fresh DEM F12)

Metabolite	concentration
Isoleucine	0.41
Leucine	1.52
Valine	1.54
Alanine	7.17
Lysine	1.64
Acetate	0,90
Glutamate/Pyroglutamate	1.24
Glutamine	3.32
Pyruvate	0.36
Succinate	1.44
Methionine	0.29
Creatine	0.86
Choline	1.21
Methanol	0.023
Glycine	0.72

Threonine	0.42
Tyrosine	0.39
Π-methylhistidine	0.11
Phenylalanine	0.56
Formate	0.03
Unknown	0.009
Glucose	17.08
Lactate	1.83
Pyroglutamate	0.77
